# Structure-Function Analysis of Volatile (*Z*)-3-Fatty Alcohols in Tomato

**DOI:** 10.1007/s10886-025-01557-7

**Published:** 2025-01-24

**Authors:** Kirsten Fisher, Harshita Negi, Owen Cole, Fallon Tomlin, Qian Wang, Johannes W. Stratmann

**Affiliations:** 1 Department of Biological Sciences, University of South Carolina, Columbia, SC USA; 2 Present Address: Department of Bacteriology, University of Wisconsin, Madison, Madison, WI USA; 3 Department of Chemistry and Biochemistry, University of South Carolina, Columbia, SC USA

**Keywords:** Green leaf volatiles, Fatty alcohols, Signal transduction, Root growth, MAP kinase (MAPK), Plant communication

## Abstract

Plants emit green leaf volatiles (GLVs) in response to biotic and abiotic stress. Receiver plants perceive GLVs as alarm cues resulting in activation of defensive or protective mechanisms. While this is well documented, it is not known how GLVs are perceived by receiver cells and what the structural determinants are for GLV activity. We tested whether the carbon chain length in (*Z*)-3-fatty alcohols with four to nine carbons and the double bonds in six-carbon alcohols contribute to bioactivity. In *Solanum peruvianum* suspension-cultured cells we found that (*Z*)-3-fatty alcohols, except (*Z*)-3-butenol, induce medium alkalinization and MAP kinase phosphorylation, two signaling responses often tied to the perception of molecular patterns that function in plant immunity and resistance to herbivores. In tomato (*S. lycopersicum*) seedlings, we found that (*Z*)-3-fatty alcohols induce inhibition of root growth. In both signaling and physiological responses, (*Z*)-3-octenol and (*Z*)-3-nonenol had a higher bioactivity than (*Z*)-3-heptenol and (*Z*)-3-hexenol, with (*Z*)-3-butenol only being active in root growth assays. Bioactivity correlated not only with chain length but also with lipophilicity of the fatty alcohols. The natural GLVs (*E*)-2-hexenol and the saturated 1-hexanol exhibited a higher bioactivity in pH assays than (*Z*)-3-hexenol, indicating that the presence and position of a double bond also contributes to bioactivity. Our results indicate that perceiving mechanisms for (*Z*)-3-fatty alcohols show a preference for longer chain fatty alcohols or that longer chain fatty alcohols are more accessible to receptors.

**Supplementary Information:**

The online version contains supplementary material available at 10.1007/s10886-025-01557-7.

## Introduction

In response to various biotic and abiotic stressors, plants release a class of volatile organic compounds known as green leaf volatiles (GLVs) (Ameye et al. 2018; Engelberth 2024; Heiden et al. 2003; Matsui and Engelberth 2022). GLVs are small, 6- to 8-carbon compounds that contain an alcohol, aldehyde, or ester functional group. Released GLVs function as environmental infochemicals by alerting distal tissue of the emitter plant and neighboring receiver plants that injury has occurred (Scala et al. 2013). Many studies have shown that this GLV-mediated inter- and intra-plant communication can directly lead to the upregulation of defenses in receiver tissue (Bate and Rothstein 1998; Engelberth et al. 2013; Yamauchi et al. 2018), or prime receiver plants to have a stronger and faster defense response against biotic stressors (Ameye et al. 2015; Engelberth et al. 2004; Frost et al. 2008; Kessler et al. 2006; Su et al. 2020). Despite the robust research on the effects of GLV mediated plant communication, how GLVs are perceived by plants cells remains conjectural (Matsui and Engelberth 2022).

Currently there are no known plant proteins homologous to the odorant receptors of animals that perceive volatile compounds and in silico predictions of plant odorant binding proteins remain to be experimentally validated (Giordano et al. 2021; Loreto and D’Auria 2022). For another volatile organic compound, the plant hormone ethylene, five histidine protein kinase receptors were identified (Bleecker and Schaller 1996), which localize to endoplasmic reticulum membranes (Hall et al. 2007). The sesquiterpene β-caryophyllene binds to the nuclear transcriptional co-repressor TOPLESS, which may release repression of defense genes (Nagashima et al. 2019). Another sesquiterpene, (-)-germacrene D, is involved in petunia flower development and perceived by an intracellular KAI2 receptor, similar to the smoke-derived seed-germination-inducing karrikins, which are butenolide derivatives (Guo et al. 2013; Stirling et al. 2024). Volatile methyl salicylate plays a role in systemic acquired resistance against pathogens. It binds to the salicylic acid binding protein SABP2, a methyl salicylate esterase, that converts methyl salicylate to salicylic acid, which induces defense responses (Gong et al. 2023).

In contrast, no GLV-binding receptor is known that mediates responses to GLVs. Some authors suggested that GLV metabolism is part of the sensing mechanism (Matsui 2016; Sugimoto et al. 2014). E.g., the green leaf alcohol (Z)-3-hexenol is glycosylated by a uridine diphosphate-glycosyltransferase (UGT91) resulting in the defense compound (*Z*)-3-hexenyl vicianoside (Sugimoto et al. 2023). Vicianoside takes 0.5–1 h to accumulate in (Z-)-3-hexenol exposed tomato plants (Sugimoto et al. 2014). While this can explain defensive functions of GLVs, metabolization cannot explain the early signaling responses that have been measured within seconds to minutes of GLV perception (Tanarsuwongkul et al. 2024). Alternatively, GLVs could be perceived by signal transducing receptors that function like classical pattern recognition receptors (PRRs) (Wang and Erb 2022). GLVs can be characterized as damage-associated molecular patterns (DAMPs) (Duran-Flores and Heil 2016; Meents and Mithofer 2020). Like DAMPs, GLVs are rapidly released from damaged plant tissue (Matsui and Engelberth 2022), and they induce some of the same early signaling events as DAMPs and other molecular patterns (Tanarsuwongkul et al. 2024), such as membrane depolarization (Zebelo et al. 2012), proton and Ca^2+^ fluxes, accumulation of γ-amino butyric acid (Asai et al. 2009; Jiao et al. 2022; Mirabella et al. 2008; Tanarsuwongkul et al. 2024; Zebelo et al. 2012) and phosphorylation of MAP kinases (Dombrowski and Martin 2018; Tanarsuwongkul et al. 2024). A direct phosphoproteomic comparison between the phytocytokine DAMP systemin and the GLVs (Z)-3-hexenol and (Z)-3-hexenyl acetate showed overlaps in rapid phosphorylation patterns in tomato cells, including phosphorylation of PRRs (Tanarsuwongkul et al. 2024). In addition, (Z)-3-hexenol, (Z)-3-hexenyl acetate, and some molecular patterns induce root growth inhibition (Holton et al. 2007; Huang et al. 2023; Tanarsuwongkul et al. 2024). Taken together, it is possible that GLVs are perceived by PRRs embedded in the plasma membrane. Alternatively, GLVs could be perceived by intracellular proteins like nucleotide-binding domain leucine-rich repeat receptors (NLRs), which also initiate signaling pathways.

The presence of PPR-like GLV receptors would presuppose that there is a motif within GLVs that lends specificity for putative receptors. Structure-function studies are useful to identify such motifs and to approximate the specificity of the interaction of a ligand and its receptor. However, few structure-function studies have been performed with GLVs. Using the GLV (*Z*)-3-hexenyl acetate and structurally related esters, Heil et al. found that the ability of these volatiles to induce extrafloral nectar (EFN) secretion in lima beans did not depend on the position or conformation of the double bond nor the size of the acid moiety (Heil et al. 2008). The apparent lack of structural effect on activity (EFN secretion) led the authors to propose an unspecific sensing mechanism for volatiles based on physiochemical processes at the plasma membrane: aliphatic GLVs dissolve in the plasma membrane and interact with and modulate various membrane proteins, leading to changes in the membrane potential which initiates signaling responses.

In their structure-activity study, Heil et al. (2008) did not investigate the impact of increasing the hydrocarbon chain length of the hexenol esters on EFN secretion. However, two other studies addressed this. Yamauchi et al. (2015) found that treatment of Arabidopsis with the reactive (*E*)-2-hexenal GLV and some (*E*)-2-aldehydes with shorter or longer chain lengths (C2 to C10) led to the accumulation of abiotic stress responsive heat shock transcription factor A2 (HSFA2). Intriguingly, the (*E*)-2-aldehydes were perceived in a chain length dependent manner with (*E*)-2-octenal being most active and (*E*)-2-decenal being largely inactive. Sugimoto et al. (2015) found that volatile (*Z*)-3-fatty alcohols [(*Z*)-3-FAlcs] ranging from 5 to 9 carbons in length ((*Z*)-2-pentenol, (*Z*)-3-hexenol, (*Z*)-3-heptenol, (*Z*)-3-octenol, and (*Z*)-3-nonenol) were able to be taken up and metabolized into their respective glucosides in Arabidopsis (Sugimoto et al. 2015). Thus, for GLV derivatives with either more or less carbons than the C6-GLV aldehydes or alcohols, there is metabolic and transcriptional evidence that they can be taken up, perceived, and metabolized by receiver plants.

The apparent structure-activity relationship for C2 to C10 (*E*)-2-aldehydes in inducing gene expression (Yamauchi et al. 2015) points to the possibility of receptors with a preference range for chain length. In contrast, a refined structure-activity analysis of GLV derivatives with different chain lengths, and double-bond positions in maize found relatively strict requirements for activating the transcription of three marker genes (Tanaka et al. 2023).

Here, we tested the ability of volatile (*Z*)-3-FAlcs of increasing chain lengths to induce DAMP-like early signaling responses in cell cultures of the wild tomato species *Solanum peruvianum* (SP cells), and a physiological effect on root growth in tomato seedlings (*S. lycopersicum*). We found that (*Z*)-3-FAlc activity depends on the carbon chain length; FAlcs with 8 and 9 carbons were more active inducers of signaling and growth responses than FAlcs with 4, 6, and 7 carbons.

## Methods and Materials

### Chemicals and Stock Solutions

The stock solutions for the (Z)-3-alcohols, (Z)-3-buten-1-ol (Z3-4OL, 98%; thermo scientific), (Z)-3-hexen-1-ol (Z3-6OL, 98%; Acros Organics), (E)-2-hexen-1-ol (96%; Acros Organics) (Z)-3-hepten-1-ol (Z3-7OL, 97%; Alfa Aesar), (Z)-3-octen-1-ol (Z3-8OL, 95%; TCI), and (Z)-3-nonen-1-ol (Z3-9OL, 97%; Alfa Aesar), and for 1-hexanol (99%; Acros Organics) were solved in 10 µL ethanol (100%, Decon Labs, Inc.) for pH and MAPK activity experiments. The stock solutions of peptide elicitor systemin (GenScript) was solved in water.

### Suspension-Cultured Cells and Plants

Heterotrophic *Solanum peruvianum* suspension-cultured cells (SP cells) were originally generated as described (Tanarsuwongkul et al. 2024). They are a long-standing, reliable, and confirmed system to measure rapid responses to extracellular signals (see Results). SP cells were grown in 125-mL Erlenmeyer flasks in MS medium on an orbital shaker (175 rpm) at 22 °C under variable room light conditions. Cells were subcultured every 12 days and used for experiments 10 days after subculturing. Tomato seedlings (*S. lycopersicum* var. Better Boy) were used for root growth assays on agar plates and grown in AR66L growth chambers (Percival Scientific) on a 16 h light (130 ± 20 µE m-2 s-1; 27 °C) and 8 h dark (22 °C) cycle. The domestic tomato *S. lycopersicum*, *S. peruvianum* and other ‘wild tomato’ species form a closely related monophyletic clade within the genus Solanum (Särkinen et al. 2013).

### pH Assay

Fatty alcohol-induced changes in the medium pH were measured as described (Stratmann et al. 2000; Tanarsuwongkul et al. 2024). Briefly, SP cells (1.5 mL) were transferred into wells of 12-well tissue culture plates (Avantor) and shaken (175 rpm) on an orbital shaker under ambient conditions. After a 1 h adjustment period, the cells were treated with a final concentration of 10 nM systemin (positive control), various concentrations of fatty alcohols solved in 10 µL ethanol, and 10 µL ethanol alone. After treatment, the medium pH was monitored using a pH probe (Mettler Toledo). To prevent cross-contamination by volatilized fatty alcohols, experimental and control treatments were each performed on separate plates.

For experiments aimed at testing the sensitivity to the proton ATPase activator fusicoccin after fatty alcohol treatment, we added the fatty alcohol or controls first (time = -5 min) and measured the induced pH change 5 min later (time = 0 min), followed by treatment with 2.5 µM fusicoccin and pH measurements over the next 45 min (time = 5–45 min).

### MAPK Phosphorylation Assay

MAPK assays were performed as described (Hann et al. 2014; Tanarsuwongkul et al. 2024). Briefly, proteins from SP cells treated with a final concentration of 3 nM systemin, 1% ethanol, and various concentrations of fatty alcohols were extracted as described (Holley et al. 2003). 20 µg of protein (15-well gels; Bio-Rad Mini-PROTEAN tetra system) was analyzed for MAPK phosphorylation by SDS PAGE followed by immunoblotting using the primary antibody anti-pERK MAPK (Phospho-p44/p42 MAPK, ERK1/2,Thr202/Tyr204, D13.14.4E; Cell Signaling Technology, Danvers, MA, USA) at a dilution of 1:2,500 and the secondary antibody (Goat anti-rabbit IgG (H + L)-HRP conjugate; Bio-Rad) at a 1:20,000 dilution, followed by a chemiluminescence assay with the Clarity™ Western ECL substrate (Bio-Rad). Membranes were stained with Coomassie brilliant blue to ensure equal protein transfer and loading. Phosphorylation signals for MPK3 and MPK4 were measured using ImageJ (Rasband, W.S., ImageJ, U. S. National Institutes of Health, Bethesda, Maryland, USA, https://imagej.net/ij/, 1997–2018.)

### Root Growth Assay

Tomato seeds were prepared as described (Tanarsuwongkul et al. 2024). In brief, they were sterilized and transferred to 1% agar containing half-strength Murashige Skoog (MS) basal medium, 0.5% (w/v) sucrose, and 0.1% (v/v) MS vitamins. Seeds on plates were cold-stratified for 2 days (4 °C) in the dark and then moved to the growth chamber and allowed to germinate for 2 days in the dark, followed by 1 day under a 16 h light (130 ± 20 µE m-2 s-1; 27 °C) and 8 h dark (22 °C) regime. On the fourth day, the seedlings were transferred to vertically oriented ½ MS agar plates and exposed to FAlcs in a 3.2 L sealed transparent plastic box. FAlcs were either pipetted on lids of 1.5 ml reaction tubes or on Q-tips attached to the lid of the box. After 24 h, the increase in root length was measured.

### Evaporation Tests

We noticed that higher volumes of C7 to C9 FAlcs applied to the incubation boxes for root growth assays did not completely evaporate within the 24-hour incubation time. Henry’s gas law, vapor pressure, and boiling point characterize the volatility of liquids. The Henry’s law constants for FAlcs are as follows: 3.72 for cis-3-butenol, 3.65 for cis-3-hexenol; 3.57 for cis-3-heptenol; 3.15 for cis-3-octenol; 3.33 for cis-3 nonenol (calculated with ChemDraw 21.0.0.). Also, the vapor pressure decreases from hexenol to nonenol from ~ 1 mm HG at 25 °C for hexenol to 0.58 mm HG for heptenol, 0.16 mm HG for octenol, and 0.07 mm Hg for nonenol, while boiling points (at 760 mm Hg) increase from 114 °C (butenol) to 202 °C (nonenol). Therefore, the volatility of fatty alcohols decreases with increasing chain length.

To determine the amount of airborne FAlcs in the incubation box, we performed an evaporation test of pure (95–98%) FALcs by measuring the amount that did not evaporate within 24 h. The weight of the FAlc solution that did not volatilize was determined by mimicking the experimental conditions (minus the seedlings) and weighing the remaining fatty alcohol solution that did not volatilize. Three independent measurements were performed to obtain the average and SD for volatilized fatty alcohols shown in the table below. Because of incomplete evaporation of higher concentrations of some (*Z*)-3-FAlcs, we showed the amount that transitioned into the gas phase over the 24 h incubation period in Figure 5.

**Table:** 

Fatty alcohol	concentration applied [µM]	Concentration in gas phase [µM ± SD]
(*Z*)-3-butenol	300	300 ± 0
(*Z*)-3-hexenol*	75	75 ± 0
	100	100 ± 0
	150	128 ± 6.7
(*Z*)-3-heptenol **	12.5	12.5 ± 0
	50	31 ± 0.03
	75	35 ± 0.03
(*Z*)-3-octenol	0.5	0.5 ± 0
	5	4.1 ± 0.22
	12.5	8.3 ± 0.04
(*Z*)-3-nonenol	1	0.9 ± 0.19
	5	2.5 ± 0.09
	12.5	3.0 ± 0.04

*(*Z*)-3-Hexenol evaporation was reported in (Tanarsuwongkul et al. 2024)

** For (*Z*)-3-heptenol: We found that after 4 h, application of 75 µM (*Z*)-3-heptenol resulted in a higher evaporation than 50 µM. When 50 µM were applied, of the 31 µM evaporated after 24 h, about 32% (10 µM) was evaporated after 4 h. When 75 µM were applied, of the 35 µM evaporated after 24 h, about 54% (19 µM) was evaporated after 4 h. This indicates a volume- or surface-dependent evaporation rate for (*Z*)-3-heptenol. Therefore, in root growth assays, saturating concentrations in the gas phase were probably reached earlier when seedlings were incubated with 75 µM (*Z*)-3-heptenol than with 50 µM, and thus seedlings were exposed for a longer time to the saturating concentration

## Results

### (Z)-3-Fatty Alcohols Induce Alkalinization of the Growth Medium of SP Cells in a Carbon Chain Length-Dependent Manner

SP cells provide a unique opportunity to study perception and signaling of structurally diverse molecular patterns. SP cells and another tomato cell line were instrumental in the discovery of the intensively studied bacterial peptide flg22 (Felix et al. 1999) and other MAMPs and DAMPs like the fungal sterol ergosterol (Granado et al. 1995), fungal chitin (Felix 1993), and RALF peptides (Pearce 2001). SP cells were also used for studies that characterized systemin binding sites (Meindl et al. 1998; Scheer and Ryan 1999), to reveal rapid changes in the phosphoproteome in response to systemin perception (Ahmad 20), and to study MAPK signaling in response to multiple structurally diverse molecular patterns (Holley et al. 2003; Stratmann et al. 2000) and UV-B radiation (Yalamanchili and Stratmann 2002). Therefore, SP cells are a versatile system to investigate structure-function correlations of FAlcs. We had shown previously that (*Z*)-3-hexenol induced a medium alkalinization response in suspension-cultured *S. peruvianum* (*SP*) cells at concentrations of > 5 mM (Tanarsuwongkul et al. 2024). While these concentrations are much higher than concentrations GLV reach when naturally emitted by plants into the surrounding airspace (see Discussion), we also showed that aerial application of (*Z*)-3-hexenol induced root growth inhibition in tomato (*S. lycopersicum*) seedlings at micromolar concentrations. Responses to (*Z*)-3-hexenol in SP cells showed a concentration-dependence, and the response was specific when compared to the GLV (*Z*)-3-hexenyl acetate, which induced medium acidification in SP cells at sub-millimolar concentrations (Tanarsuwongkul et al. 2024). Therefore, the pH response is a convenient assay to determine structure-activity relationships among comparable FAlcs.

**Fig. 1:**
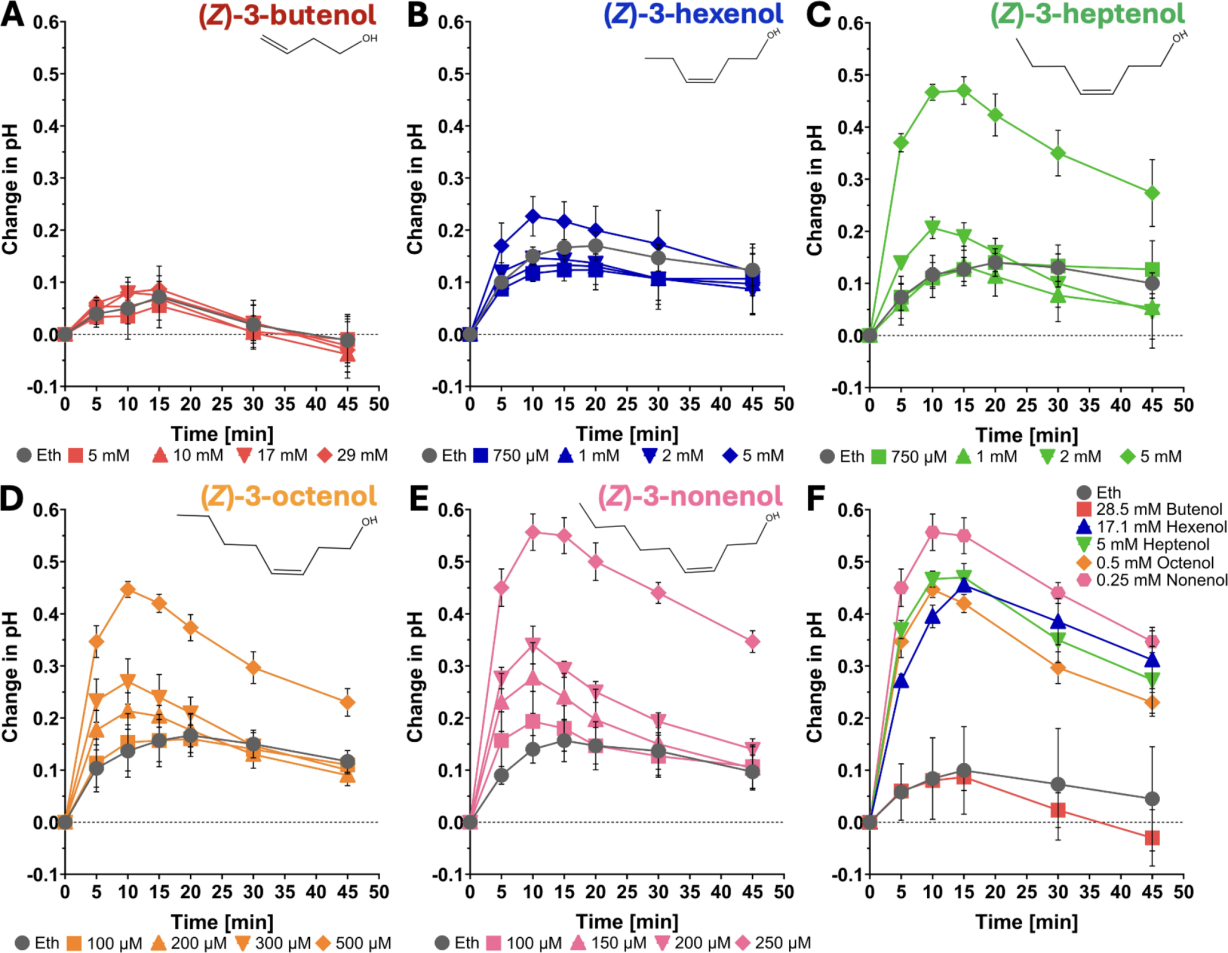
Kinetics of medium pH changes elicited by (*Z*)-3-FAlcs in SP cells. (**A-E**) SP cells were treated with various concentrations of (*Z*)-3-FAlcs solved in 10 µL ethanol as indicated and 10 µL ethanol alone. The medium pH was measured over a period of 45 min. The change in medium pH is expressed as compared to the pH at time = 0. Graphs represent the average and standard deviations of three independent experiments (*n* = 3). Concentrations that elicited a pH response above the ethanol control were determined through a two-way ANOVA followed by a Holm‐Šídák multiple comparisons test. (**F**) For a direct comparison, concentrations of (*Z*)-3-FAlcs are shown that elicit a pH change of 0.5 ± 0.1 within 15 min. For statistical details, see Supp. Table 1. In summary, pH changes in (*Z*)-3-butenol and (*Z*)-3-hexenol-treated cells were not significantly exceeding ethanol controls, whereas (*Z*)-3-heptenol was significantly active above ethanol controls at ≥ 2 mM, (*Z*)-3-octenol at ≥ 200 µM, (*Z*)-3-nonenol at ≥ 150 µM

We focused on (*Z*)-3-FAlcs with different chain lengths ranging from four (C4) to nine carbons (C9). We were unable to obtain (*Z*)-3-pentenol (C5) and (*Z*)-3-FAlcs with more than 9 carbons. To determine the activity of (*Z*)-3-FAlcs, we compared the pH response of SP cells induced by FAlcs to the response to the solvent control ethanol. SP cells responded to (*Z*)-3-FAlcs with a medium alkalinization response, except for (*Z*)-3-butenol (C4) (Fig. 1A-F). (*Z*)-3-hexenol at concentrations of ≤ 5 mM was not active (although, for 5 mM, the P value was 0.048, but only at the 10 min time point; Supp. Table 1), indicating that the lowest active concentration is slightly above 5 mM. We had shown earlier that it is active at concentrations of ≥ 10 mM (Tanarsuwongkul et al. 2024). In contrast, the longer-chain FAlcs were more active, with (*Z*)-3-heptenol (C7) being active at ≥ 2 mM, (*Z*)-3-octenol (C8) at ≥ 200 µM, and (*Z*)-3-nonenol (C9) at ≥ 150 µM (Supp. Table 1). Figure 1F illustrates the concentrations of each FAlc necessary to induce a medium alkalinization response of 0.5 ± 0.1 pH units, consistent with the structure-activity relationship observed in Fig. 1A–E, where longer-chain FAlcs induce the pH change at lower concentrations compared to shorter-chain FAlcs. which follows the same structure-activity pattern as shown in Fig. 1A-E with longer FAlcs inducing the pH change at lower concentrations than shorter FAlcs. When comparing all FAlcs at a concentration of 5 mM, we again see the same dependence of activity on chain length (Supp. Figure 1). A corresponding ANOVA analysis shows that, at 5 mM, (*Z*)-3-heptenol is more active than (*Z*)-3-butenol and (*Z*)-3-hexenol, which are inactive at 5 mM, and (*Z*)-3-octenol and (*Z*)-3-nonenol are more active than (*Z*)-3-heptenol at all time points from 5 to 45 min. The activity of (*Z*)-3-octenol and (*Z*)-3-nonenol was similar, and (*Z*)-3-nonenol was significantly different from (*Z*)-3-octenol only at 5 min after application. Taken together, these results suggest that the carbon chain length of FAlcs is a critical factor influencing their perception by SP cells.

### The Presence and Position of a Double Bond in Six-Carbon FAlcs Have an Effect on the Medium Alkalinization Response in SP Cells

**Fig. 2:**
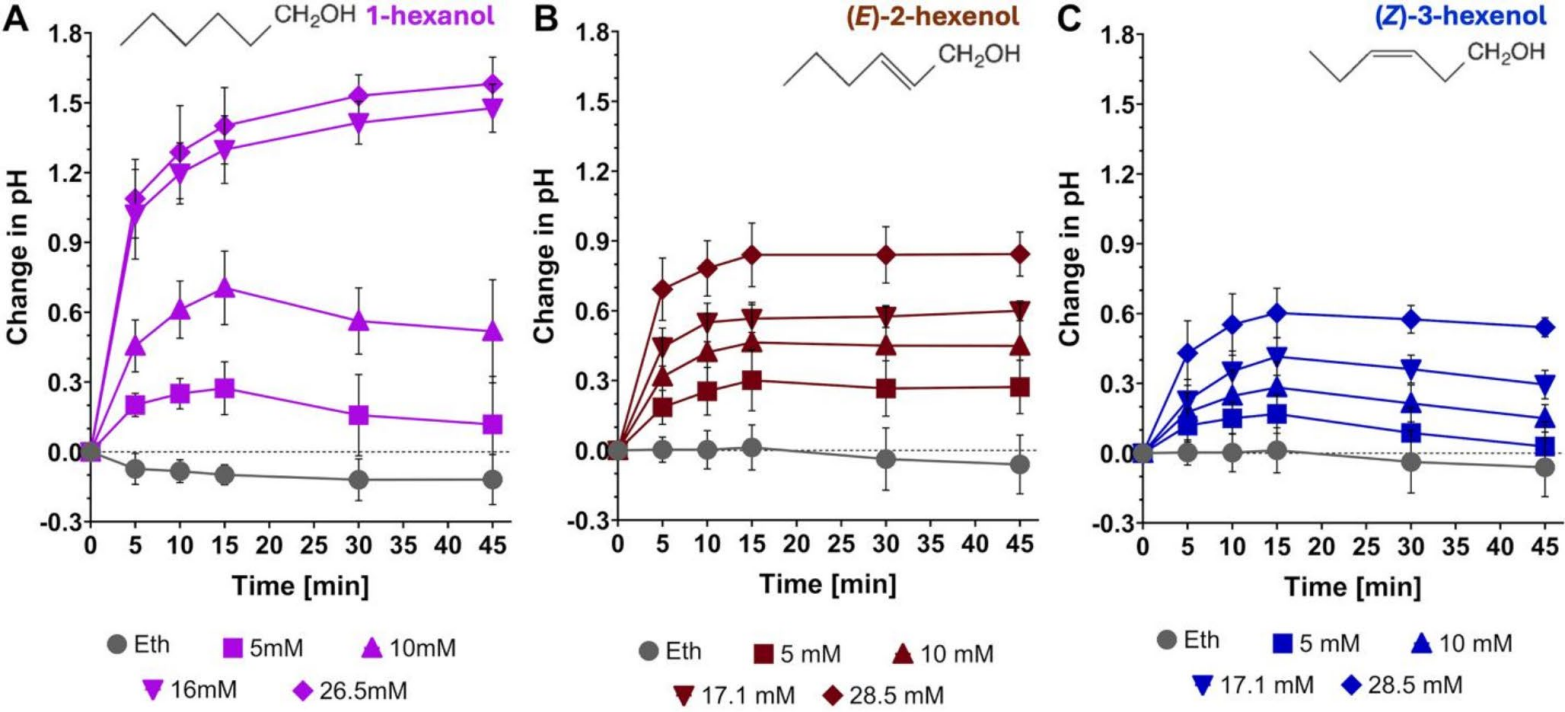
Kinetics of medium pH changes elicited by 1-hexanol (**A**), (*E*)*-2*-hexenol (**B**), and (*Z*)-3-hexenol (**C**). SP cells were treated with various concentrations of GLVs solved in 10 µL ethanol as indicated and 10 µL ethanol alone. The medium pH was measured over a period of 45 min. The change in medium pH is expressed as compared to the pH at time = 0. Graphs represent the average and standard deviations of three independent experiments (*n* ≥ 3). Concentrations that elicited a pH response above the ethanol control were determined through a two-way ANOVA followed by a Holm‐Šídák multiple comparisons test. For statistical details, see Supp. Table 2. In summary, (*E*)*-2*-hexenol and 1-hexanol were significantly active above ethanol controls at ≥ 5 mM, whereas P-values for 5 mM (*Z*)-3-hexenol at 10 and 15 min were barely < 0.05, and > 0.05 at the other time points Notice: (*E*)*-2*-Hexenol and (*Z*)-3-hexenol were tested in the same experiments with the same ethanol controls and the same batch of SP cells. Results for (*Z*)-3-hexenol were published previously (Tanarsuwongkul et al. 2024) under a Creative Commons open access license (CC BY 4.0). The exact same data are shown here again (**C**) but adjusted to the format of this figure, and the corresponding statistics are shown in Supp. Tables 2, to facilitate comparison of the three GLVs tested

We also compared whether the presence of the double bond is important for bioactivity of six-carbon alcohols. Saturated 1-hexanol was more active than (*Z*)-3-hexenol. Ten mM 1-hexanol induced a response comparable to 28.5 mM of (*Z*)-3-hexenol, while at 5 mM, 1-hexanol was weakly but significantly active and (*Z*)-3-hexenol was inactive (Fig. 2A, C**)**. A concentration of 16 mM 1-hexanol elicited a substantially stronger response than 10 mM, with no further increase observed at 28.5 mM. In addition, (*E*)*-2*-hexenol induced a stronger alkalinization response than (*Z*)-3-hexenol, but a weaker response than 1-hexanol (Fig. 2), with a concentration of 17.1 mM (*E*)*-2*-hexenol inducing a response comparable to 28.5 mM (*Z*)-3-hexenol and 10 mM 1-hexanol.

### The Response of FAlc-treated SP Cells to the Proton ATPase Activator Fusicoccin Depends on the Chain Lengths of the FAlcs

The alkalinization response is a common response to many elicitors of plant defenses (Boller and Felix 2009) and most likely a consequence of ion fluxes and inhibition of plasma membrane proton ATPase activity (Ahmad et al. 2019; Falhof et al. 2016; Schaller and Oecking 1999). Active proton ATPases charge the proton gradient across the membrane, leading to a near neutral pH in the cytosol and an acidic pH in the apoplast, which translates to a pH of 4 to 5 in the growth medium of SP cells. In the case of FAlcs, another scenario for the alkalinization response is that the lipophilic FAlcs disturb the integrity of the plasma membrane (Sikkema et al. 1995). This could result in a proton ATPase-independent extracellular alkalinization via diffusion of protons along the proton gradient into the cell.

This effect should be more pronounced for longer, more lipophilic FAlcs. To test whether the plasma membrane is still capable of maintaining or establishing a proton gradient after FAlc treatment, we treated SP cells with the fungal toxin and proton ATPase agonist fusicoccin. Fusicoccin locks the proton ATPase in an active state inducing an extracellular acidification and thus hypercharging the proton gradient (Falhof et al. 2016). In contrast, if membrane integrity is disturbed, fusicoccin-activated proton ATPase activity would not result in reestablishing the proton gradient.

**Fig. 3:**
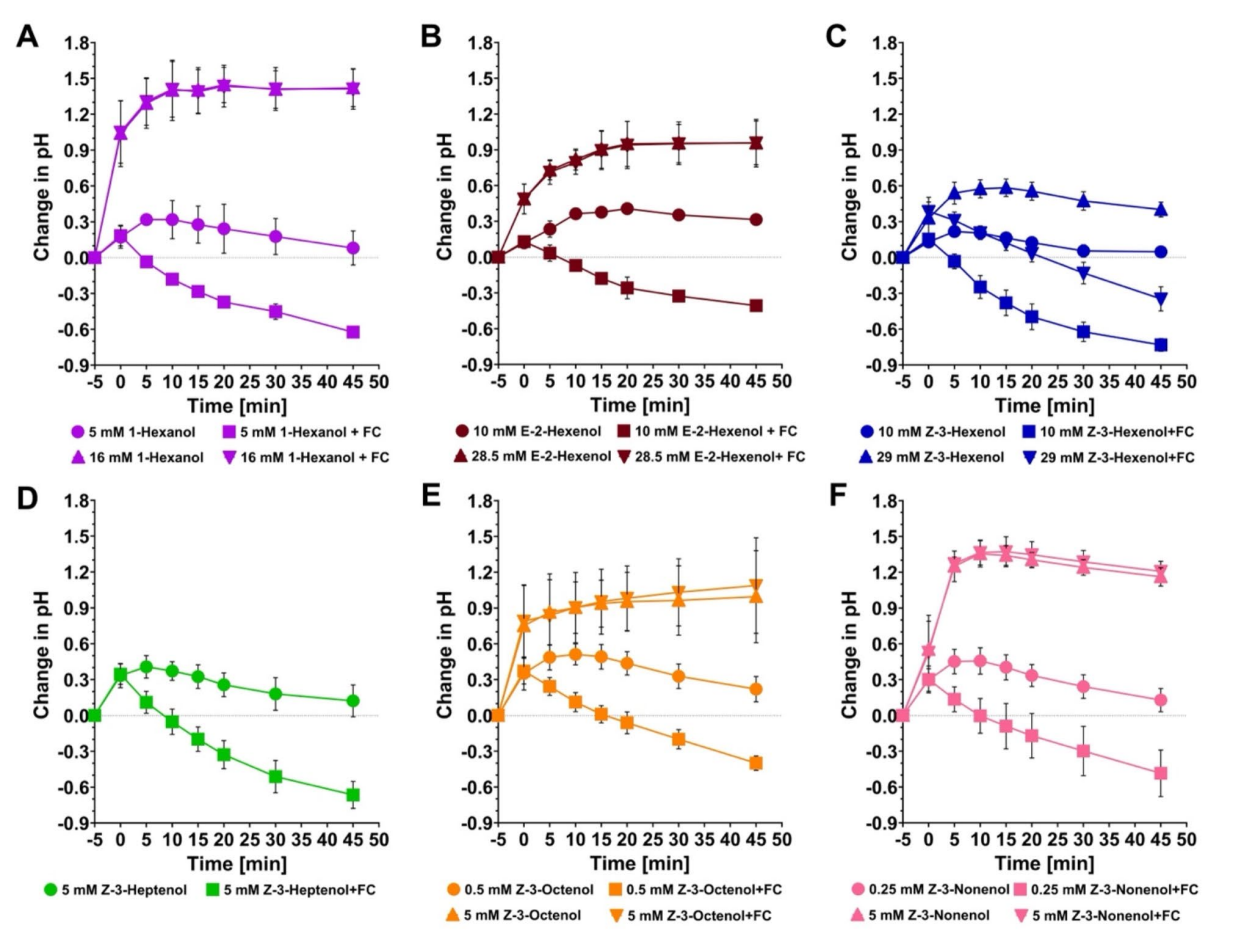
Fusicoccin re-establishes the proton gradient across the plasma membrane after treatment with FAlcs. (**A– F**). SP cells were treated with either 10 µL ethanol or FAlcs at the concentrations indicated at t = -5 min. Five minutes later (t = 0), cells were either treated with 2.5 µM fusicoccin (FC) or not, and the medium pH was recorded over 45 min. Graphs represent the average and standard deviations of three independent experiments (*n* = 3). A two-way ANOVA followed by a Holm‐Šídák multiple comparisons test was performed to determine whether responses to the FAlcs alone are significantly different from responses to FAlcs plus fusicoccin. For statistical details, see Supp. Table 3. In summary, significant differences were determined for 5 mM 1-hexanol, 10 mM (*E*)*-2*-hexenol, 10 and 28.5 mM (*Z*)-3-hexenol, 5 mM (*Z*)-3-heptenol, 0.5 mM (*Z*)-3-octenol, and 0.25 mM (*Z*)-3-nonenol

To demonstrate the fusicoccin effect in a well-established system, we treated SP cells with systemin to cause a medium alkalinization response. Five minutes later, cells were treated with fusicoccin. Fusicoccin immediately blocked a further systemin-induced medium alkalinization as seen in controls and induced a rapid and strong acidification response over the next 45 min, similar to what we had shown earlier (Higgins et al. 2007). Comparing the response of untreated and ethanol-treated cells showed that ethanol does not alter the response of SP cells to fusicoccin (Supp. Figure 2). When SP cells were treated with C6-C9 FAlcs, we observed that fusicoccin can acidify the medium in cells treated with 10 and 28.5 mM (*Z*)-3-hexenol, 5 mM (*Z*)-3-heptenol, 0.5 mM (*Z*)-3-octenol, and 0.25 mM (*Z*)-3-nonenol. Fusicoccin applied 5 min after (*Z*)-3-octenol and (*Z*)-3-nonenol at concentrations of 5 mM did not induce an acidification response, indicating that, at those concentrations, these longer-chain (*Z*)-3-FAlcs interfere with the process that establishes the proton gradient across the plasma membrane (Fig. 3). In two of three experiments, fusicoccin induced medium acidification after a 1 mM (*Z*)-3-octenol treatment, but not after a 0.5 mM (*Z*)-3-nonenol treatment, indicating that (*Z*)-3-nonenol has a stronger membrane disruption effect than (*Z*)-3-octenol (preliminary experiments; not shown in Fig. 3). In our previous work, we had shown that fusicoccin can induce medium acidification when applied 90 min after treatment of SP cells with 28.5 mM (*Z*)-3-hexenol (Tanarsuwongkul et al. 2024). When applied 5 min after 28.5 mM (*Z*)-3-hexenol, fusicoccin also induced a medium acidification (Fig. 3), indicating that hexenol does not interfere with membrane integrity even at high concentrations.

We also tested whether cells treated with 1-hexanol and (*E*)*-2*-hexenol would respond to fusicoccin (Fig. 3A, B). 1-Hexanol triggered a pronounced alkalinization response at 16 and 26.5 mM, but SP cells exposed to 16 mM 1-hexanol were unresponsive to fusicoccin, whereas cells treated with 5 mM 1-hexanol exhibited medium acidification in response to fusicoccin. Similarly, SP cells treated with 28.5 mM (*E*)*-2*-hexenol did not respond to fusicoccin, whereas cells treated with 5 mM (*E*)*-2*-hexenol did. This indicates that the high concentrations of (*Z*)-3-nonenol, (*Z*)-3-octenol, 1-hexanol and (*E*)*-2*-hexenol either disturbed the integrity of the membrane or directly interfered with ion flux mechanisms or proton ATPase activity.

### (Z)-3-Fatty Alcohols Induce MAP Kinase Phosphorylation in SP Cells in a Carbon Chain Length-Dependent Manner

**Fig. 4:**
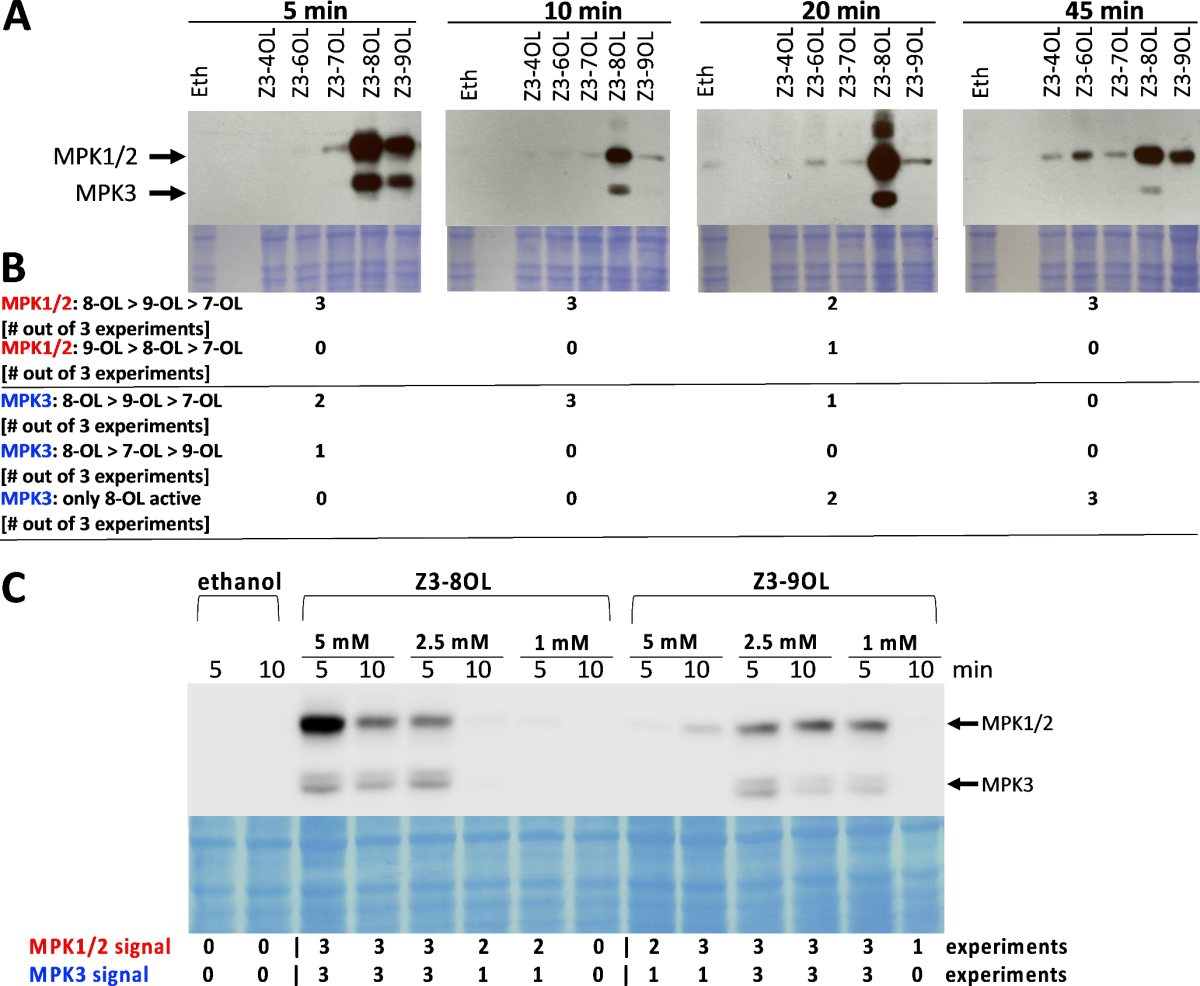
Comparison of MAPK phosphorylation in response to (*Z*)-3-FAlcs. (**A**) SP cells were treated for 5–45 min with 5 mM (*Z*)-3-butenol, -hexenol, -heptenol, and -octenol (Z3-4-OL to Z3-9-OL) that were solved in 10 µL ethanol, and with 10 µL ethanol (Eth) alone. Phosphorylation of MAPKs was visualized by immunoblotting using an antibody against the phosphorylated MAPK activation motif pTEpY. Coomassie-stained membranes are shown to demonstrate equal loading and transfer. One of three independent experiments is shown (for all experiments, see Supp. Figure 3). (**B**) MAPK phosphorylation signals in response to the five (*Z*)-3-FAlcs were compared by experiment based on quantification of MPK1/2 and MPK3 signals as explained in Methods and Materials. (**C**) MAPK phosphorylation induced by 1 to 5 mM (*Z*)-3-octenol and (*Z*)-3-nonenol. SP cells were treated for 5 and 10 min with either 5 mM, 2.5 mM, or 1 mM of (*Z*)-3-octenol or (*Z*)-3-nonenol, or with 10 µL ethanol. Phosphorylation of MAPKs was determined as described under (A). One experiment and the corresponding coomassie-stained membrane is shown. Results of all three independent experiments are shown as described under (B). All experiments are shown in Supp. Figure 4

We showed earlier that the GLV (*Z*)-3-hexenol induced phosphorylation of mitogen-activated protein kinases (MAPKs) in SP cells (Tanarsuwongkul et al. 2024). Here we compared the MAPK response in SP cells to (*Z*)-3-FAlcs with different chain lengths, ranging from four (C4) to nine carbons (C9), at a concentration of 5 mM. We found that (*Z*)-3-butenol and (*Z*)-3-hexenol are largely inactive at this concentration over a time course of 45 min. In contrast, the longer chain (*Z*)-3-FAlcs triggered the phosphorylation of the tomato MAPKs MPK1/2 and MPK3 (Fig. 4A). MPK1 and MPK2 are 95.4% identical at the amino acid level (Holley 03). Due to their nearly identical molecular mass (45.5 and 45.2 kD), they cannot be distinguished by size on an immunoblot. In contrast, the smaller MPK3 (42.8 kD) can be distinguished from MPK1 and MPK2.

(*Z*)-3-Octenol and (*Z*)-3-nonenol induced stronger MPK1/2 phosphorylation than (*Z*)-3-heptenol in 3 out of 3 independent experiments at all four time points tested (Fig. 4A, B; Supp. Figure 3). (*Z*)-3-octenol was more active than (*Z*)-3-nonenol, except for one experiment at one time point (20 min). MPK3 phosphorylation increased in response to (*Z*)-3-heptenol and (*Z*)-3-nonenol at 5 min, increased weakly in response to (*Z*)-3-nonenol at 10 min, and in response to (*Z*)-3-octenol at all times (Fig. 4A, B; Supp. Figure 3). (*Z*)-3-hexenol induced MPK1/2 and MPK3 phosphorylation at a concentration of 28.5 mM (Tanarsuwongkul et al. 2024). Preliminary experiments indicated that (*Z*)-3-hexenol was also active at 17 mM (not shown). At 5 mM, it was not significantly active, but it induced weak MPK1/2 phosphorylation at each time point in one of the three independent experiments, indicating that 5 mM may be close to the lowest active (*Z*)-3-hexenol concentration (Fig. 4A; Supp. Figure 3).

Since (*Z*)-3-octenol and (*Z*)-3-nonenol induced strong MPK1/2 phosphorylation at 5 mM, we also tested lower concentrations. At 5 min, 1 mM (*Z*)-3-octenol induced MPK1/2 phosphorylation in 2 of 3 independent experiments, and at 2.5 mM in 3 of 3 experiments (Fig. 4C and Supp. Figure 4). At 10 min, 1 mM (*Z*)-3-octenol was inactive, but at 2.5 mM it was active in 2 of 3 experiments. In contrast, 1 mM (*Z*)-3-nonenol induced MPK1/2 activity in 3 of 3 experiments at 5 min and in one experiment at 10 min. At 2.5 mM, it induced MPK1/2 phosphorylation in all experiments at 5 and 10 min. The effects of the two FAlcs on MPK3 phosphorylation were similar to MPK1/2. This indicates that (*Z*)-3-nonenol may be slightly more active than (*Z*)-3-octenol at lower concentrations. In the same experiments, 5 mM concentrations reproduced the effect shown in Fig. 4A. We also tested 0.5 mM for octenol and nonenol, but in only one of three experiments we observed a MAPK response to both FAlcs at 5 min and no response at 10 min (not shown in figure). So, while (*Z*)-3-octenol is more active than (*Z*)-3-nonenol at 5 mM, (*Z*)-3-nonenol is more active than (*Z*)-3-octenol at 2.5 and 1 mM, but these are relatively subtle differences. Collectively, these assays confirmed our findings from pH assays that FAlc bioactivity depends on the carbon chain length.

### (Z)-3-Fatty Alcohols Inhibit Root Growth of Tomato Seedlings in a Carbon Chain Length-Dependent Manner

**Fig. 5:**
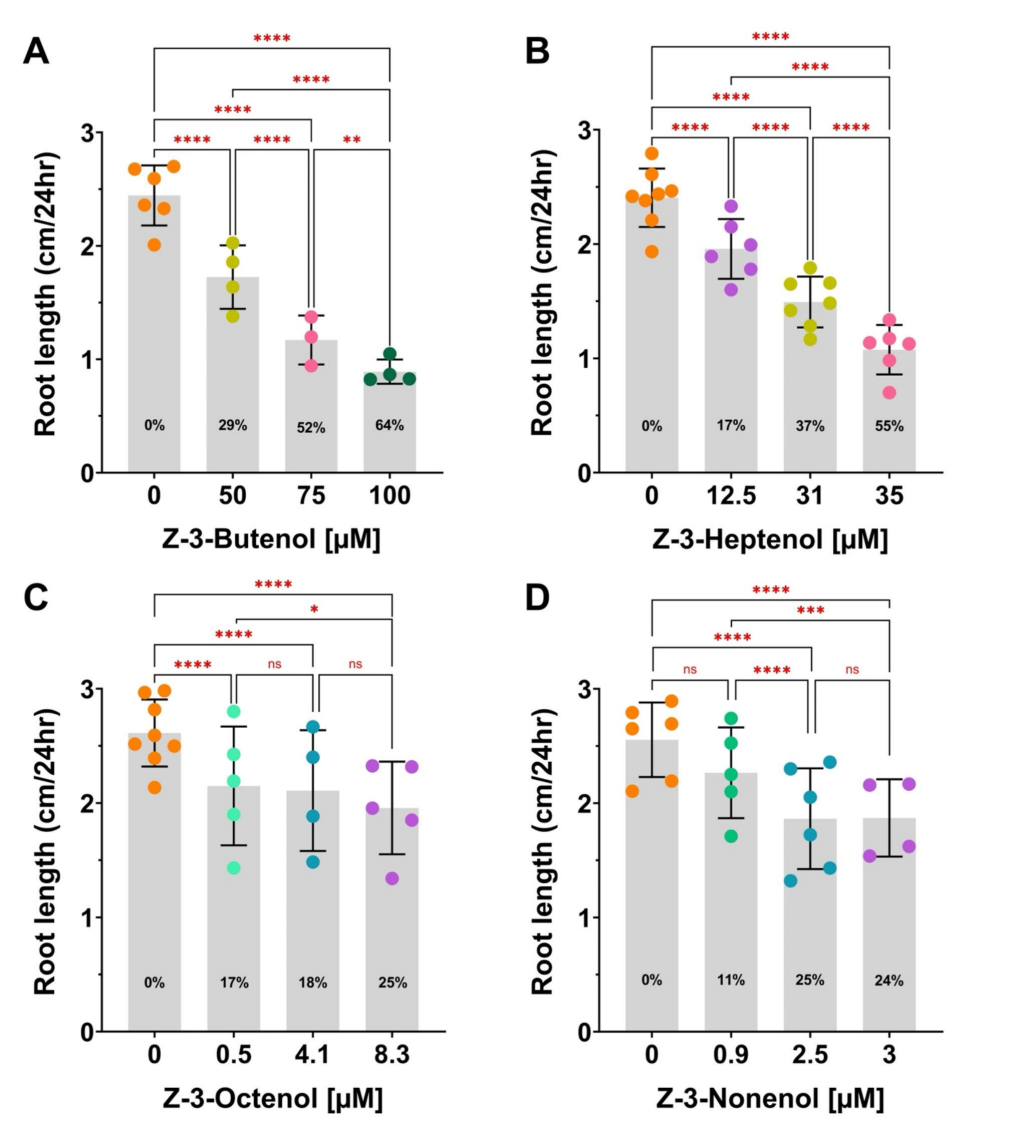
Root growth inhibition induced by FAlcs in tomato seedlings. Sterile stratified seeds were incubated for three days in a growth chamber (16:8 h light: dark), then placed on top of vertically oriented agar plates and incubated for 24 h in a sealed box with either (*Z*)-3-butenol (**A**), (*Z*)-3-heptenol (**B**), (*Z*)-3-octenol (**C**), or (*Z*)-3-nonenol (**D**) at the concentrations indicated. Bars show averages of increases in root length over 24 h. Averages represent 3 to 8 independent experiments (shown as dots) and SD with a sample size of 12 per experiment. Percentages in bars refer to percent root growth inhibition compared to untreated controls. Significant differences between treated and untreated (0 µM) plants were determined through a nested one-way ANOVA followed by a Holm‐Šídák multiple comparisons test (****p ≤ 0.0001; *** *p* = 0.003; **p = 0.0052; *p = 0.0437; ns—not significant, *p* ≥ 0.05)

To test whether the structure-activity relationships of (*Z*)-3-FAlcs tested in SP suspension-cultured cells hold for applications of FAlcs to plants in the gas phase, we tested the effect of various (*Z*)-3-FAlcs on the root growth of tomato (*S. lycopersicum*) seedlings. Root growth assays are frequently used to test perception of elicitors of plant defenses. Many molecular patterns, e.g., flg22 and systemin (Gomez-Gomez et al. 1999; Holton et al. 2007), induce root growth inhibition. This probably reflects a growth-defense trade-off when plants reduce growth in favor of defense (He et al. 2022; Smakowska et al. 2016). We had shown earlier that (*Z*)-3-hexenol induces root growth inhibition in this system (Tanarsuwongkul et al. 2024). For root growth assays, seeds were stratified, germinated, and placed on top of vertically oriented agar plates, followed by incubation with (*Z*)-3-FAlcs for 24 h in sealed transparent boxes in a growth chamber with a 16 h light and 8 h dark cycle. We found that the evaporation of longer-chain FAlcs under our conditions is incomplete at higher concentrations and determined the concentration of the FAlcs in the gas phase, as described in Methods and Materials. Since we showed the amount of FAlcs that transitioned into the gas phase over the 24 h incubation period in Fig. 5, the numbers for the C7-C9 FAlcs may appear arbitrary. Higher concentrations in the gas phase cannot be reached for (*Z*)-3-octenol and (*Z*)-3-nonenol than the ones we tested. Therefore, the maximal bioactivity is limited by physical properties of the FAlcs.

We tested various concentrations to approximate the lowest active concentration. 50 µM (*Z*)-3-butenol induced 29% root growth inhibition on average (Fig. 5A), while 12.5 µM induced no significant inhibition (not shown in Fig. 5). This effect of (*Z*)-3-butenol on roots contrasts with its inactivity in SP cells (Figs. 1 and 4). Earlier, we showed that 75 µM (*Z*)-3-hexenol induced 19% root growth inhibition on average and lower concentrations were inactive (Tanarsuwongkul et al. 2024). Therefore, (*Z*)-3-butenol had a stronger effect on root growth than (*Z*)-3-hexenol. At a concentration of 12.5 µM, (*Z*)-3-heptenol induced 17% root growth inhibition on average (Fig. 5B), but it was inactive at 5 µM (not shown in Fig. 5). The lowest active concentration measured for (*Z*)-3-octenol was 0.5 µM (17%inhibition), but higher concentrations up to 8.3 µM did not significantly increase the effect (Fig. 5C). Concentrations higher than 8.3 µM were not tested because of physical limitations for evaporation under our conditions (see Methods and Materials– *Evaporation tests*). (*Z*)-3-Nonenol was not significantly active at 0.9 µM, but 2.5 µM induced 25% root growth inhibition. At 3 µM this effect was not further increased (Fig. 5D) and higher concentrations could not be tested due to low volatility. At the lowest active concentrations, different FAlcs induced a different maximal root growth inhibition (Table 1). (*Z*)-3-Butenol at 100 µM induced 64% root growth inhibition as compared to untreated controls. However, higher concentrations caused symptoms of necrosis (browning of cotyledons and root tips), indicating a possible toxicity effect at higher concentrations. (*Z*)-3-Hexenol induced a maximal root growth inhibition of 30% at 150 µM without any stress symptoms (Tanarsuwongkul et al. 2024), while application of 200 µM caused mild bleaching of cotyledons in some seedlings. Longer (Z)-3-FAlcs were active at significantly lower concentrations and did not induce bleaching or necrosis. Due to their physical constraints regarding volatility, active concentration ranges for octenol and nonenol were relatively narrow, ~ 8 µM for (*Z*)-3-octenol, and only ~ 1 µM for (*Z*)-3-nonenol. This contrasts with (*Z*)-3-butenol and (*Z*)-3-hexenol, which are active over a broad range of concentrations (~ 50–75 µM). (*Z*)-3-Heptenol is in between with a range of ~ 20 µM. The significant increase in root growth inhibition in seedlings treated with 31 and 35 µM (*Z*)-3-heptenol is probably not due to the final concentrations in the gas phase we determined at the end of the experiment (24 h), but rather to a higher evaporation rate for the higher concentration (see Methods and Materials).

The maximal non-toxic growth response is not dependent on the chain lengths of the FAlcs. (*Z*)-3-Hexenol is similar to (*Z*)-3-nonenol (25–30%) and (*Z*)-3-butenol is similar to (*Z*)-3-heptenol (64 and 55%, respectively) (Table 1). For all (*Z*)-3-FAlcs, we did not observe a nearly complete inhibition of root growth (≥ 90%), as shown for other factors in Arabidopsis, such as (*E*)*-2*-hexenal (Mirabella et al. 2008; Scala et al. 2017) or the molecular pattern pep1 (Huang et al. 2023). However, other molecular patterns like flg22 and nlp20 induced root growth inhibition in the range of the (*Z*)-3-FAlcs, i.e., ~ 50 or 25%, respectively (Huang et al. 2023). In summary, a clear structure-activity relationship exists for C6–C9 (*Z*)-3-FAlcs, with bioactivity increasing from (*Z*)-3-hexenol to (*Z*)-3-nonenol, except for the outlier (*Z*)-3-butenol.

**Table 1:** Summary of the (*Z*)-3-FAlc effects on root growth in tomato seedlings

	Active concentration			
	^a^lowest [µM]	^b^highest [µM]	^C^maximal inhibition	Notes
Z3-butenol	≤ 50	≥ 100, < 150	64%	≥ 150 µM toxic
Z3-hexenol	≤ 75	≥ 150, < 200	30%	≥ 200 µM toxic
Z3-heptenol	≤ 12.5	≥ 35	55%	> 75 µM not tested
Z3-octenol	≤ 0.5	≥ 8.3	25%	no additional effect > 0.5 µM
Z3-nonenol	≤ 2.6	≥ 3	25%	no additional effect > 2.6 µM

^a^ Concentration at which the lowest significant root growth inhibition was observed (see Fig. 5), with lower concentrations possibly still inducing a significant effect. ^b^ Concentration that caused the strongest observed root growth inhibition with higher concentrations possibly increasing the effect. ^c^ Maximal induced root growth inhibition as percentage of root growth in untreated controls (= 0%)

## Discussion

Structure-function analyses for putative ligands of unknown receptors have the potential to reveal structural determinants that affect the response to that ligand. By testing rapid signaling responses to structurally related ligands, differences in these responses are most likely due to differential interactions of these ligands with a cognate receptor. We tested whether the carbon chain length and its saturation would affect two rapid signaling responses in suspension-cultured SP cells. We found that three structural properties of FAlcs determine their bioactivity, saturation (weakly), carbon chain length, and lipophilicity. The two latter properties also affected the activity in root growth assays, which test effects of airborne FAlcs on a slow physiological response. In our previous work, we showed that the responses to (*Z*)-3-hexenol and (*Z*)-3-hexenyl acetate, which contain the same carbon chain, are different with regard to pH, MAPK, root growth, and phosphoproteome response (Tanarsuwongkul et al. 2024). A possible conclusion is that the carbon chain enables interaction of a FALc with a receptor protein, while the functional group is shaping the specificity of the response. Longer carbon chains would then facilitate the FAlc-receptor interaction leading to a stronger response.

It is worth noting that the natural GLVs 1-hexanol, (*Z*)-3-hexenol, and (*E*)-2-hexenol show less activity in our assays than the corresponding FAlcs with 7 to 9 carbon chains, which, to our knowledge, are not known to be emitted by plants. However, plants are known to emit other longer chain volatile alcohols and respond to them, e.g. pathogen-induced 1-octen-3-ol and 3-octanol (Kishimoto et al. 2007; Song et al. 2022). Fungi emit 1-octen-3-ol as well (Schenkel et al. 2015). These natural volatiles may bind to the same or similar receptors as GLVs. Also, there is precedence from a study on hydroxy fatty acids supporting the idea that the active motif for GLVs could be the length of its carbon chain, which would determine the affinity to receptors. In Arabidopsis the extracellular lectin receptor kinase LORE perceives medium chain 3-hydroxy fatty acids in a chain length dependent manner, with 3-hydroxydecanoic acid (3-OH-C10:0) eliciting a stronger immune response than 3-hydroxy fatty acids with 8, 9, and 11 to 16 carbons (Kutschera et al. 2019). In the end, we cannot exclude that FAlc perception in tomato may be unspecific and only determined by lipophilicity. This scenario, however, would not explain the specific signaling effects known to be induced by GLVs and longer chain FAlcs.

The C6-FAlcs 1-hexanol, (*Z*)-3-hexenol, and (*E*)*-2*-hexenol are natural GLVs synthesized in response to cell damage from linoleic or linolenic acid through the lipoxygenase and hydroperoxide lyase pathway (Scala et al. 2013; Selman et al. 2024). We found that all three C6-FAlcs induce a rapid alkalinization response in SP cells (Fig. 2). Based on fusicoccin experiments (Fig. 3), we conclude that 1-hexanol and (*E*)*-2*-hexenol were slightly more active than (*Z*)-3-hexenol. Therefore, the position and the presence of the double bond in C6-GLVs affect the bioactivity slightly but the response to fusicoccin strongly, and thus how they interact with the plasma membrane.

When comparing the pH response to (*Z*)-3-FAlcs with different carbon chain lengths, a longer carbon chain length correlated with a stronger alkalinization response in SP cells (Fig. 1; Table 1). In addition, (*Z*)-3-octenol and (*Z*)-3-nonenol at concentrations of ≥ 5 mM induced responses that could not be reverted by fusicoccin (Fig. 3), indicating an unspecific effect on the plasma membrane. In contrast, lower concentrations of these FAlcs did not interfere with fusicoccin (Fig. 3). The fusicoccin experiments indicate that the pH response to (*Z*)-3-FAlcs has two components, one being unspecific, presumably through disturbance of membrane integrity, and one being specific.

For the latter, we hypothesize that the response is mediated by a FAlc receptor and contributes to signal transduction in a similar way as extracellular alkalinization and MAPK activation induced by molecular patterns like flg22 or systemin (Felix and Boller 1995; Felix et al. 1999; Hann et al. 2014; Holley et al. 2003; Stratmann et al. 2000). In support of this hypothesis, Ahmad et al. (2019) had shown in SP cells that plasma membrane proton ATPase activity is rapidly reduced in response to systemin and that this correlated with a specific systemin-induced phosphorylation pattern of two tomato proton ATPases (AHA1 and LHA1). In our previous work, we found that (*Z*)-3-hexenol also induced rapid AHA1 dephosphorylation of two key phosphosites, presumably leading to AHA1 inhibition (Falhof et al. 2016; Tanarsuwongkul et al. 2024). However, we cannot exclude other specific effects of (*Z*)-3-FAlcs, e.g., on transporters, ion channels, or ion pumps that regulate ion homeostasis across the plasma membrane and thus the extracellular pH.

When we extended our structure-function analysis of (*Z*)-3-FAlcs to their potential for MAPK activation, we found a similar correlation of bioactivity and carbon chain length as for the pH response (Fig. 4, Supp. Figure 3, Supp. Figure 4, Table 2). Considering unspecific effects due to interference of FAlcs with membrane integrity as determined by our fusicoccin experiments, the results from MAPK assays largely confirm results from pH assays. However, for (*Z*)-3-octenol and (*Z*)-3-nonenol, the lowest active concentrations were slightly above fusicoccin sensitivity, as determined by pH assays. Therefore, we cannot exclude that MAPK activation by these two FAlcs could partly be due to membrane disturbance effects. On the other hand, we are not aware of a mechanism that would link membrane disturbance to MAPK activation, which generally requires the entire MAPK cascade to be activated by an intra- or extracellular receptor. We had shown earlier that MAPK activation and changes in extracellular pH are not dependent of each other (Higgins et al. 2007), e.g., fusicoccin and systemin both activate the same MAPKs in SP cells but have opposite effects on proton fluxes, inducing either medium acidification or alkalinization, respectively.

MAPKs respond rapidly to many stress signals, including molecular patterns, often within 5 min after treatment (Holley et al. 2003; Kandoth et al. 2007). They are typically activated when molecular patterns bind to PRRs or when effector proteins from pathogens are detected by intracellular NLR-receptors (Zhang and Zhang 2022). Thus, activation by GLVs of the same MAPKs that are involved in molecular pattern and effector signaling, usually the orthologs of the Arabidopsis MAPKs MPK6, MPK4, and MPK3 (Zhang and Zhang 2022), lends credence to the idea that GLVs are perceived like molecular patterns or effectors (Tanarsuwongkul et al. 2024).

The interaction of FAlcs with plant cells is clearly different in suspension-cultured cells and in cells within an intact organism. High concentrations of FAlcs solved in liquid growth medium are required to induce signaling responses in SP cells, whereas lower concentrations of airborne FAlcs are sufficient to induce a growth response in intact plants. This indicates that interactions of FAlcs with SP cells are reduced, possibly through partial aggregation of FAlcs (Sassi et al. 2004). Solubility is probably not a key factor. Solubility decreases with increasing chain length from (*Z*)-3-butenol to (*Z*)-3-octenol (from 109 g/L to 1.9 g/L), but at the concentrations applied in our assays FAlcs should be soluble in an aqueous solution, unless they aggregate. In intact plants, whether FAlcs interact with an extracellular domain of a membrane bound receptor or diffuse through the plasma membrane, they would first have to pass the cell wall area with its aqueous environment and presumably also become solubilized (Widhalm et al. 2015).

When FAlcs reach the plasma membrane, lipophilicity plays an important role for how the FAlcs interact with cells. For plants exposed to airborne volatile compounds, this is also an important factor (Camacho-Coronel et al. 2020; Heil et al. 2008), especially for larger compounds. The possible disturbance of membrane integrity by higher concentrations of the C7-C9 FAlcs in our suspension-cultured SP cells correlates with their log*P* numbers (n-octanol-water partition coefficient), which provide information on the lipophilicity of compounds. The (*Z*)-3-isomers of butenol, hexenol, heptenol, octenol, and nonenol have log*P* numbers of 0.6 to 2.9, as shown in Table 2. To provide a reference, methanol has a log*P* number of -0.5, and the sesquiterpene germacrene D has a log*P* of 4.7. For the (*Z*)-3-FAlcs, considering activity in pH and MAPK assays as well as their effects on fusicoccin, we found a clear lipophilicity-bioactivity relationship. The higher the lipophilicity (higher log*P* numbers), the stronger the biological response. Since longer FAlcs exhibit higher lipophilicity, the lipophilicity-bioactivity and the structure-bioactivity relationships overlap (Table 2). Consequently, the greater activity of longer-chain FAlcs (C7–C9) compared to six-carbon FAlcs may be attributed to their structural and physical properties, which likely enhance their access to perception mechanisms.

We also explored whether the structure-activity correlation for signaling responses in SP cells would be reproducible when young tomato seedlings are exposed to FAlcs in the gas phase. By and large, the effects of (*Z*)-3-FAlcs on seedlings and SP cells are similar.

However, there are some notable differences. First, in seedlings, concentrations that induced a response were much lower than in SP cells, e.g., for (*Z*)-3-octenol and (*Z*)-3-nonenol 400- and 60-fold, respectively. Second, (*Z*)-3-butenol was more active than (*Z*)-3-hexenol in seedlings but inactive in SP cells. Third, in root growth assays, (*Z*)-3-octenol was slightly more active than (*Z*)-3-nonenol, but it was the opposite in SP cell assays. Overall, the bioactivity of these two FAlcs in both seedlings and SP cells is notably similar and significantly greater than that of the shorter-chain FAlcs.

**Table 2:** pH and MAPK effects of FAlcs compared to logP numbers and sensitivity to fusicoccin

Chemical	logP ^a^PC/^b^GSC	pH response lowest active concentration [mM]	MAPK response lowest active concentration [mM]	fusicoccin sensitivity [mM]	^c^Rank
Z-3-butenol	0.6/0.7	^d^inactive	^e^inactive/> 5	^f^n.t.	7
Z-3-hexenol	1.3/1.7	> 5	> 5	≤ 28.5	6
E-2-hexenol	1.4/1.7	≤ 5	n.t.	≤ 10	5/4
1-hexanol	2/2	≤ 5	n.t.	≤ 5	5/4
Z-3-heptenol	1.9/2.2	2	5	≤ 5	3
Z-3-octenol	2.4/2.7	0.2	≥ 1	≤ 0.5	2
Z-3-nonenol	2.9/3.2	0.15	≤ 1	≤ 0.25	1

^a^ PC = PubChem (https://pubchem.ncbi.nlm.nih.gov/*);* PC uses calculated “XLogP3” numbers (Cheng et al. 2007)

^b^ GSC = The Good Scents Company (http://www.thegoodscentscompany.com/index.html*);* generally calculated (“estimated”) numbers, sometimes using XLogP3 like PubChem; GSC does not reveal alternative calculation for logP numbers

^**c**^ Rank regarding overall bioactivity in pH and MAPK assays with 7 for lowest and 1 for highest activity

^d^ inactive at concentrations up to 28.5 mM (see Fig. 2)

^e^ inactive at 5 mM; concentrations > 5 mM not tested

^f^ n.t. - not tested

We do not know whether the concentrations we tested in our artificial system for measuring effects of FAlcs on root growth are physiologically relevant. In the field, concentrations of volatile organic compounds in the air are variable due to plume formations during emission bouts (Beyaert and Hilker 2014), reaching temporarily high concentrations. In this study, we are interested in whether signaling and physiological responses depend on the chain length of (Z)-3-FAlcs, and our data clearly demonstrate that they do. Regardless of the nature of the unknown receptor or perception mechanism for (*Z*)-3-hexenol in tomato, it is unlikely to interact exclusively with (*Z*)-3-hexenol.

Our findings are strikingly different from another structure-activity study of FAlcs. Tanaka et al. (2023) tested the effect of various FAlcs and other GLV-related derivatives on gene expression in two-week-old maize seedlings by spraying 1 mM solutions of test compounds onto leaves, or by exposing them to test compounds in the gas phase using an airflow system. They found that only (*Z*)-3- and (*E*)-3-hexenol induced a response, while n-hexanol, (*Z*)-3-heptenol, (*Z*)-3-octenol, and (*Z*)-3-nonenol were inactive and did not induce gene expression. (*Z*)-2-Pentenol, (*Z*)-4-heptenol, and (*Z*)-5-octenol, FAlcs that we did not analyze, were also inactive. The differences between this study with a monocot plant and our study with a dicot plant may reflect a taxonomy-specific difference for perception and activity of FAlcs. Another factor could be the nature of the response. Stress gene expression is induced via signaling pathways, and while we examined signaling components, we did not investigate direct downstream effects of signaling, such as gene expression. It is conceivable that altered proton flux dynamics and MAPK activity are not involved in regulating the expression of tomato genes that are orthologous to the (*Z*)-3- and (*E*)-2-hexenol-responsive genes in maize tested in the Tanaka et al. (2023) study. But the two MAPKs we observed in the tomato system, MPK1/2 and MPK3, play a role in systemin-induced herbivory-related gene expression (Kandoth et al. 2007). Responses to molecular patterns or DAMPs often follow a structure-activity pattern showing an optimal structural feature, e.g., amino acid sequence in peptides (Felix et al. 1999; Pearce 1993) or carbon chain length in 3-hydroxy fatty acids (Kutschera et al. 2019). On the other hand, the sesquiterpene (-)-germacrene D is stereospecifically perceived by the receptor KAI2 in petunia flowers (Stirling et al. 2024) while (+)-germacrene D was inactive. It will be an exciting research direction to explore how the structure of GLVs determines their perception in a broader taxonomic and biochemical context in future studies.

## Electronic Supplementary Material

Below is the link to the electronic supplementary material.

Supplementary Material 1

Supplementary Material 2

## Data Availability

No datasets were generated or analysed during the current study.

## References

[CR1] Ahmad FH, Wu XN, Stintzi A, Schaller A, Schulze WX (2019) The Systemin Signaling Cascade as derived from time course analyses of the systemin-responsive Phosphoproteome. Mol Cell Proteom 18:1526–1542. 10.1074/mcp.RA119.001367 10.1074/mcp.RA119.001367 PMC6683004 31138643

[CR3] Ameye M, Audenaert K, De Zutter N, Steppe K, Van Meulebroek L, Vanhaecke L, De Vleesschauwer D, Haesaert G, Smagghe G (2015) Priming of wheat with the Green Leaf volatile Z-3-Hexenyl acetate enhances defense against Fusarium Graminearum but boosts Deoxynivalenol Production. Plant Physiol 167:1671–1684. 10.1104/pp.15.00107 25713338 10.1104/pp.15.00107 PMC4378182

[CR2] Ameye M, Allmann S, Verwaeren J, Smagghe G, Haesaert G, Schuurink RC, Audenaert K (2018) Green leaf volatile production by plants: a meta-analysis. New Phytol 220:666–683. 10.1111/nph.14671 28665020 10.1111/nph.14671

[CR4] Asai N, Nishioka T, Takabayashi J, Furuichi T (2009) Plant volatiles regulate the activities of Ca2+ -permeable channels and promote cytoplasmic calcium transients in Arabidopsis leaf cells. Plant Signal Behav 4:294–300. 10.4161/psb.4.4.8275 19794844 10.4161/psb.4.4.8275 PMC2664488

[CR5] Bate NJ, Rothstein SJ (1998) C-volatiles derived from the lipoxygenase pathway induce a subset of defense-related genes. Plant J 16:561–569. 10.1046/j.1365-313x.1998.00324.x 10036774 10.1046/j.1365-313x.1998.00324.x

[CR6] Beyaert I, Hilker M (2014) Plant odour plumes as mediators of plant–insect interactions. Biol Rev 89:68–81. 10.1111/brv.12043 23714000 10.1111/brv.12043

[CR7] Bleecker AB, Schaller GE (1996) The mechanism of Ethylene Perception. Plant Physiol 111:653–660. 10.1104/pp.111.3.653 12226320 10.1104/pp.111.3.653 PMC157880

[CR8] Boller T, Felix G (2009) A Renaissance of elicitors: Perception of Microbe-Associated molecular patterns and Danger signals by pattern-recognition receptors. Annu Rev Plant Biol 60:379–406. 10.1146/annurev.arplant.57.032905.105346 19400727 10.1146/annurev.arplant.57.032905.105346

[CR9] Camacho-Coronel X, Molina-Torres J, Heil M (2020) Sequestration of exogenous volatiles by Plant Cuticular Waxes as a mechanism of Passive Associational Resistance: a proof of Concept. Frontiers in plant science 11. 10.3389/fpls.2020.00121 10.3389/fpls.2020.00121 PMC7052286 32158455

[CR10] Cheng T, Zhao Y, Li X, Lin F, Xu Y, Zhang X, Li Y, Wang R, Lai L (2007) Computation of octanol– water partition coefficients by guiding an additive model with knowledge. J Chem Inf Model 47:2140–2148. 10.1021/ci700257y 17985865 10.1021/ci700257y

[CR11] Dombrowski JE, Martin RC (2018) Activation of MAP kinases by green leaf volatiles in grasses. BMC Res Notes 11:79. 10.1186/s13104-017-3076-9 29378628 10.1186/s13104-017-3076-9 PMC5789745

[CR12] Duran-Flores D, Heil M (2016) Sources of specificity in plant damaged-self recognition. Curr Opin Plant Biol 32:77–87. 10.1016/j.pbi.2016.06.019 27421107 10.1016/j.pbi.2016.06.019

[CR13] Engelberth J (2024) Green Leaf volatiles: a New Player in the Protection against Abiotic stresses? Int J Mol Sci 25:9471. 10.3390/ijms25179471 39273416 10.3390/ijms25179471 PMC11395555

[CR14] Engelberth J, Alborn HT, Schmelz EA, Tumlinson JH (2004) Airborne signals prime plants against insect herbivore attack. Proc Natl Acad Sci USA 101:1781–1785 14749516 10.1073/pnas.0308037100 PMC341853

[CR15] Engelberth J, Contreras CF, Dalvi C, Li T, Engelberth M (2013) Early transcriptome analyses of Z-3-hexenol-treated zea mays revealed distinct transcriptional networks and anti-herbivore defense potential of green leaf volatiles. PLoS ONE 8:e77465. 10.1371/journal.pone.0077465 24155960 10.1371/journal.pone.0077465 PMC3796489

[CR16] Falhof J, Pedersen JT, Fuglsang AT, Palmgren M (2016) Plasma membrane H(+)-ATPase regulation in the Center of Plant Physiology. Mol Plant 9:323–337. 10.1016/j.molp.2015.11.002 26584714 10.1016/j.molp.2015.11.002

[CR17] Felix G, Boller T (1995) Systemin induces rapid ion fluxes and ethylene biosynthesis in *Lycopersicon peruvianum* cells. Plant J 7:381–389

[CR19] Felix G, Regenass M, Boller T (1993) Specific perception of subnanomolar concentrations of chitin fragments by tomato cells: induction of extracellular alkalinization, changes in protein phosphorylation, and establishment of a refractory state. Plant J 4:307–316

[CR18] Felix G, Duran JD, Volko S, Boller T (1999) Plants have a sensitive perception system for the most conserved domain of bacterial flagellin. Plant J 18:265–276 10377992 10.1046/j.1365-313x.1999.00265.x

[CR20] Frost CJ, Mescher MC, Carlson JE, De Moraes CM (2008) Plant defense priming against herbivores: getting ready for a different battle. Plant Physiol 146:818–824. 10.1104/pp.107.113027 18316635 10.1104/pp.107.113027 PMC2259053

[CR21] Giordano D, Facchiano A, D’Auria S, Loreto F (2021) A hypothesis on the capacity of plant odorant-binding proteins to bind volatile isoprenoids based on in silico evidences. Elife 10. 10.7554/eLife.66741 10.7554/eLife.66741 PMC8221805 34161230

[CR22] Gomez-Gomez L, Felix G, Boller T (1999) A single locus determines sensitivity to bacterial flagellin in *Arabidopsis thaliana*. Plant J 18:277–284. 10.1046/J.1365-313x.1999.00451.X 10377993 10.1046/j.1365-313x.1999.00451.x

[CR23] Gong Q, Wang Y, He L, Huang F, Zhang D, Wang Y, Wei X, Han M, Deng H, Luo L, Cui F, Hong Y, Liu Y (2023) Molecular basis of methyl-salicylate-mediated plant airborne defence. Nature 622:139–148. 10.1038/s41586-023-06533-3 37704724 10.1038/s41586-023-06533-3

[CR24] Granado J, Felix G, Boller T (1995) Perception of fungal sterols in plants. Plant Phys 107:485–490 10.1104/pp.107.2.485 PMC157151 12228375

[CR25] Guo Y, Zheng Z, La Clair JJ, Chory J, Noel JP (2013) Smoke-derived karrikin perception by the α/β-hydrolase KAI2 from Arabidopsis. Proc Natl Acad Sci 110:8284–8289. 10.1073/pnas.1306265110 23613584 10.1073/pnas.1306265110 PMC3657771

[CR26] Hall BP, Shakeel SN, Schaller GE (2007) Ethylene receptors: Ethylene Perception and Signal Transduction. J Plant Growth Regul 26:118–130. 10.1007/s00344-007-9000-0

[CR27] Hann CT, Bequette CJ, Dombrowski JE, Stratmann JW (2014) Methanol and ethanol modulate responses to danger- and microbe-associated molecular patterns. Front Plant Sci 5:550. 10.3389/fpls.2014.00550 25360141 10.3389/fpls.2014.00550 PMC4197774

[CR28] He Z, Webster S, He SY (2022) Growth-defense trade-offs in plants. Curr Biol 32:R634–R639. 10.1016/j.cub.2022.04.070 35728544 10.1016/j.cub.2022.04.070

[CR29] Heiden AC, Kobel K, Langebartels C, Schuh-Thomas G, Wildt J (2003) Emissions of oxygenated volatile organic compounds from plants - part I: emissions from lipoxygenase activity. J Atmos Chem 45:143–172. 10.1023/a:1024069605420

[CR30] Heil M, Lion U, Boland W (2008) Defense-inducing volatiles: in search of the active motif. J Chem Ecol 34:601–604. 10.1007/s10886-008-9464-9 18408973 10.1007/s10886-008-9464-9 PMC2373414

[CR31] Higgins R, Lockwood T, Holley S, Yalamanchili R, Stratmann J (2007) Changes in extracellular pH are neither required nor sufficient for activation of mitogen-activated protein kinases (MAPKs) in response to systemin and fusicoccin in tomato. Planta 225:1535–1546 17109147 10.1007/s00425-006-0440-8

[CR32] Holley SR, Yalamanchili RD, Moura SD, Ryan CA, Stratmann JW (2003) Convergence of signaling pathways induced by systemin, oligosaccharide elicitors, and ultraviolet-B radiation at the level of mitogen-activated protein kinases in *Lycopersicon peruvianum* suspension-cultured cells. Plant Physiol 132:1728–1738 12913131 10.1104/pp.103.024414 PMC181261

[CR33] Holton N, Cano-Delgado A, Harrison K, Montoya T, Chory J, Bishop GJ (2007) Tomato *BRASSINOSTEROID INSENSITIVE1* is required for Systemin-Induced Root Elongation in *Solanum pimpinellifolium* but is not essential for Wound Signaling. Plant Cell 19:1709–1717. 10.1105/tpc.106.047795 17513502 10.1105/tpc.106.047795 PMC1913732

[CR34] Huang Y, Cui J, Li M, Yang R, Hu Y, Yu X, Chen Y, Wu Q, Yao H, Yu G, Guo J, Zhang H, Wu S, Cai Y (2023) Conservation and divergence of flg22, pep1 and nlp20 in activation of immune response and inhibition of root development. Plant Sci 331:111686. 10.1016/j.plantsci.2023.111686 36963637 10.1016/j.plantsci.2023.111686

[CR35] Jiao C, Guo Z, Gong J, Zuo Y, Li S, Vanegas D, McLamore ES, Shen Y (2022) CML8 and GAD4 function in (Z)-3-hexenol-mediated defense by regulating gamma-aminobutyric acid accumulation in Arabidopsis. Plant Physiol Biochem 186:135–144. 10.1016/j.plaphy.2022.06.023 35842997 10.1016/j.plaphy.2022.06.023

[CR36] Kandoth PK, Ranf S, Pancholi SS, Jayanty S, Walla MD, Miller W, Howe GA, Lincoln DE, Stratmann JW (2007) Tomato MAPKs LeMPK1, LeMPK2, and LeMPK3 function in the systemin-mediated defense response against herbivorous insects. PNAS 104:12205–12210. 10.1073/pnas.0700344104 17623784 10.1073/pnas.0700344104 PMC1924534

[CR37] Kessler A, Halitschke R, Diezel C, Baldwin IT (2006) Priming of plant defense responses in nature by airborne signaling between Artemisia tridentata and Nicotiana attenuata. Oecologia 148:280–292. 10.1007/s00442-006-0365-8 16463175 10.1007/s00442-006-0365-8

[CR38] Kishimoto K, Matsui K, Ozawa R, Takabayashi J (2007) Volatile 1-octen-3-ol induces a defensive response in Arabidopsis thaliana. J Gen Plant Pathol 73:35–37. 10.1007/s10327-006-0314-8

[CR39] Kutschera A, Dawid C, Gisch N, Schmid C, Raasch L, Gerster T, Schäffer M, Smakowska-Luzan E, Belkhadir Y, Vlot AC, Chandler CE, Schellenberger R, Schwudke D, Ernst RK, Dorey S, Hückelhoven R, Hofmann T, Ranf S (2019) Bacterial medium-chain 3-hydroxy fatty acid metabolites trigger immunity in *Arabidopsis* plants. Science 364:178–181. 10.1126/science.aau1279 30975887 10.1126/science.aau1279

[CR40] Leal WS (2013) Odorant reception in insects: roles of receptors, binding proteins, and degrading enzymes. Ann Rev Entomol 58:373–391. 10.1146/annurev-ento-120811-153635 23020622 10.1146/annurev-ento-120811-153635

[CR41] Loreto F, D’Auria S (2022) How do plants sense volatiles sent by other plants? Trends Plant Sci 27:29–38. 10.1016/j.tplants.2021.08.009 34544607 10.1016/j.tplants.2021.08.009

[CR42] Matsui K (2016) A portion of plant airborne communication is endorsed by uptake and metabolism of volatile organic compounds. Curr Opin Plant Biol 32:24–30. 10.1016/j.pbi.2016.05.005 27281633 10.1016/j.pbi.2016.05.005

[CR43] Matsui K, Engelberth J (2022) Green Leaf Volatiles-the Forefront of Plant Responses against Biotic Attack. Plant Cell Physiol 63:1378–1390. 10.1093/pcp/pcac117 35934892 10.1093/pcp/pcac117

[CR44] Meents AK, Mithofer A (2020) Plant-Plant Communication: Is There a Role for Volatile Damage-Associated Molecular Patterns? Frontiers in plant science 11:583275. 10.3389/fpls.2020.583275 10.3389/fpls.2020.583275 PMC7593327 33178248

[CR45] Meindl T, Boller T, Felix G (1998) The plant wound hormone systemin binds with the N-terminal part to its receptor but needs the C-terminal part to activate it. Plant Cell 10:1561–1570. 10.1105/tpc.10.9.1561 9724701 10.1105/tpc.10.9.1561 PMC144085

[CR46] Mirabella R, Rauwerda H, Struys EA, Jakobs C, Triantaphylides C, Haring MA, Schuurink RC (2008) The Arabidopsis her1 mutant implicates GABA in E-2-hexenal responsiveness. Plant J 53:197–213. 10.1111/j.1365-313X.2007.03323.x 17971036 10.1111/j.1365-313X.2007.03323.x

[CR47] Nagashima A, Higaki T, Koeduka T, Ishigami K, Hosokawa S, Watanabe H, Matsui K, Hasezawa S, Touhara K (2019) Transcriptional regulators involved in responses to volatile organic compounds in plants. J Biol Chem 294:2256–2266. 10.1074/jbc.RA118.005843 30593507 10.1074/jbc.RA118.005843 PMC6378981

[CR48] Pearce G, Johnson S, Ryan CA (1993) Structure-activity of deleted and substituted systemin, an 18-amino acid polypetide inducer of plant defensive genes. J Biol Chem 268:212–216 8416929

[CR49] Pearce G, Moura D, Stratmann J, Ryan CA (2001) RALF, a 5-kDa ubiquitous polypeptide in plants, arrests root growth and development. Proc Natl Acad Sci USA 98:12843–12847 11675511 10.1073/pnas.201416998 PMC60141

[CR50] Särkinen T, Bohs L, Olmstead RG, Knapp S (2013) A phylogenetic framework for evolutionary study of the nightshades (Solanaceae): a dated 1000-tip tree. BMC Evol Biol 13:214. 10.1186/1471-2148-13-214 24283922 10.1186/1471-2148-13-214 PMC3850475

[CR51] Sassi P, Paolantoni M, Cataliotti RS, Palombo F, Morresi A (2004) Water/Alcohol mixtures: a spectroscopic study of the water-saturated 1-Octanol solution. J Phys Chem B 108:19557–19565. 10.1021/jp046647d

[CR52] Scala A, Allmann S, Mirabella R, Haring MA, Schuurink RC (2013) Green Leaf volatiles: a Plant’s Multifunctional Weapon against herbivores and pathogens. Int J Mol Sci 14:17781–17811. 10.3390/ijms140917781 23999587 10.3390/ijms140917781 PMC3794753

[CR53] Scala A, Mirabella R, Goedhart J, de Vries M, Haring MA, Schuurink RC (2017) Forward genetic screens identify a role for the mitochondrial HER2 in E-2-hexenal responsiveness. Plant Mol Biol 95:399–409. 10.1007/s11103-017-0659-8 28918565 10.1007/s11103-017-0659-8 PMC5688203

[CR54] Schaller A, Oecking C (1999) Modulation of plasma membrane H^+^-ATPase activity differentially activates wound and pathogen defense responses in tomato plants. Plant Cell 11:263–272 9927643 10.1105/tpc.11.2.263 PMC144172

[CR55] Scheer J, Ryan CA (1999) A 160-kD systemin receptor on the surface of *Lycopersicon peruvianum* suspension-cultured cells. Plant Cell 11:1525–1535. 10.1105/tpc.11.8.1525 10449585 10.1105/tpc.11.8.1525 PMC144299

[CR56] Schenkel D, Lemfack MC, Piechulla B, Splivallo R (2015) A meta-analysis approach for assessing the diversity and specificity of belowground root and microbial volatiles. Front Plant Sci 6. 10.3389/fpls.2015.00707 10.3389/fpls.2015.00707 PMC4568395 26442022

[CR57] Selman S, Engelberth M, Engelberth J (2024) Organizing the Chaos: Novel insights into the regulation of Z-3-Hexenal production in damaged Maize leaves. Preprints. 10.20944/preprints202409.0176.v1 10.3390/plants13192772 PMC11479226 39409641

[CR58] Sikkema J, Bont JAd, Poolman B (1995) Mechanisms of membrane toxicity of hydrocarbons. Microbiol Rev 59:201–222. 10.1128/mr.59.2.201-222.1995 7603409 10.1128/mr.59.2.201-222.1995 PMC239360

[CR59] Smakowska E, Kong J, Busch W, Belkhadir Y (2016) Organ-specific regulation of growth-defense tradeoffs by plants. Curr Opin Plant Biol 29:129–137. 10.1016/j.pbi.2015.12.005 26802804 10.1016/j.pbi.2015.12.005

[CR60] Song GC, Jeon J-S, Choi HK, Sim H-J, Kim S-G, Ryu C-M (2022) Bacterial type III effector–induced plant C8 volatiles elicit antibacterial immunity in heterospecific neighbouring plants via airborne signalling. Plant Cell Environ 45:236–247. 10.1111/pce.14209 34708407 10.1111/pce.14209 PMC9298316

[CR61] Stirling SA, Guercio AM, Patrick RM, Huang X-Q, Bergman ME, Dwivedi V, Kortbeek RWJ, Liu Y-K, Sun F, Tao WA, Li Y, Boachon B, Shabek N, Dudareva N (2024) Volatile communication in plants relies on a KAI2-mediated signaling pathway. Science 383:1318–1325. 10.1126/science.adl4685 38513014 10.1126/science.adl4685

[CR62] Stratmann J, Scheer J, Ryan CA (2000) Suramin inhibits initiation of defense signaling by systemin, Chitosan and pmg-elicitor in suspension cultured *Lycopersicon peruvianum* cells. Proc Natl Acad Sci USA 97:8862–8867. 10.1073/pnas.97.16.8862 10922047 10.1073/pnas.97.16.8862 PMC34024

[CR63] Su Q, Yang FB, Zhang QH, Tong H, Hu Y, Zhang XY, Xie W, Wang SL, Wu QJ, Zhang YJ (2020) Defence priming in tomato by the green leaf volatile (Z)-3-hexenol reduces whitefly transmission of a plant virus. Plant Cell Environ 43:2797–2811. 10.1111/pce.13885 32955131 10.1111/pce.13885

[CR64] Sugimoto K, Matsui K, Iijima Y, Akakabe Y, Muramoto S, Ozawa R, Uefune M, Sasaki R, Alamgir KM, Akitake S, Nobuke T, Galis I, Aoki K, Shibata D, Takabayashi J (2014) Intake and transformation to a glycoside of (Z)-3-hexenol from infested neighbors reveals a mode of plant odor reception and defense. Proc Natl Acad Sci USA 111:7144–7149. 10.1073/pnas.1320660111 24778218 10.1073/pnas.1320660111 PMC4024874

[CR65] Sugimoto K, Matsui K, Takabayashi J (2015) Conversion of volatile alcohols into their glucosides in Arabidopsis. Commun Integr Biol 8:e992731. 10.4161/19420889.2014.992731 26629260 10.4161/19420889.2014.992731 PMC4594374

[CR66] Sugimoto K, Ono E, Inaba T, Tsukahara T, Matsui K, Horikawa M, Toyonaga H, Fujikawa K, Osawa T, Homma S, Kiriiwa Y, Ohmura I, Miyagawa A, Yamamura H, Fujii M, Ozawa R, Watanabe B, Miura K, Ezura H, Ohnishi T, Takabayashi J (2023) Identification of a tomato UDP-arabinosyltransferase for airborne volatile reception. Nat Commun 14:677. 10.1038/s41467-023-36381-8 36755045 10.1038/s41467-023-36381-8 PMC9908901

[CR67] Tanaka Y, Fujita K, Date M, Watanabe B, Matsui K (2023) Structure-activity relationship of volatile compounds that induce defense-related genes in maize seedlings. Plant Signal Behav 18:2234115. 10.1080/15592324.2023.2234115 37454374 10.1080/15592324.2023.2234115 PMC10730182

[CR68] Tanarsuwongkul S, Fisher KW, Mullis BT, Negi H, Roberts J, Tomlin F, Wang Q, Stratmann JW (2024) Green leaf volatiles co-opt proteins involved in molecular pattern signalling in plant cells. Plant Cell Environ 47:928–946. 10.1111/pce.14795 38164082 10.1111/pce.14795

[CR69] Wang L, Erb M (2022) Volatile uptake, transport, perception, and signaling shape a plant’s nose. Essays Biochem 66:695–702. 10.1042/ebc20210092 36062590 10.1042/EBC20210092 PMC9528081

[CR70] Wang Z-Y, Seto H, Fujioka S, Yoshida S, Chory J (2001) BRI1 is a critical component of a plasma-membrane receptor for plant steroids. Nature 410:380–383. 10.1038/35066597 11268216 10.1038/35066597

[CR71] Widhalm JR, Jaini R, Morgan JA, Dudareva N (2015) Rethinking how volatiles are released from plant cells. Trends Plant Sci 20:545–550. 10.1016/j.tplants.2015.06.009 26189793 10.1016/j.tplants.2015.06.009

[CR72] Wu F, Chi Y, Jiang Z, Xu Y, Xie L, Huang F, Wan D, Ni J, Yuan F, Wu X, Zhang Y, Wang L, Ye R, Byeon B, Wang W, Zhang S, Sima M, Chen S, Zhu M, Pei J, Johnson DM, Zhu S, Cao X, Pei C, Zai Z, Liu Y, Liu T, Swift GB, Zhang W, Yu M, Hu Z, Siedow JN, Chen X, Pei Z-M (2020) Hydrogen peroxide sensor HPCA1 is an LRR receptor kinase in Arabidopsis. Nature 578:577–581. 10.1038/s41586-020-2032-3 32076270 10.1038/s41586-020-2032-3

[CR73] Yalamanchili RD, Stratmann JW (2002) Ultraviolet-B activates components of the systemin signaling pathway in *Lycopersicon peruvianum* suspension-cultured cells. J Biol Chem 277:28424–28430 12034744 10.1074/jbc.M203844200

[CR74] Yamauchi Y, Kunishima M, Mizutani M, Sugimoto Y (2015) Reactive short-chain leaf volatiles act as powerful inducers of abiotic stress-related gene expression. Sci Rep-Uk 5:8030. 10.1038/srep08030 10.1038/srep08030 PMC4306126 25619826

[CR75] Yamauchi Y, Matsuda A, Matsuura N, Mizutani M, Sugimoto Y (2018) Transcriptome analysis of Arabidopsis thaliana treated with green leaf volatiles: possible role of green leaf volatiles as self-made damage-associated molecular patterns. J Pestic Sci 43:207–213. 10.1584/jpestics.D18-020 30363142 10.1584/jpestics.D18-020 PMC6140709

[CR76] Zebelo SA, Matsui K, Ozawa R, Maffei ME (2012) Plasma membrane potential depolarization and cytosolic calcium flux are early events involved in tomato (Solanum lycopersicon) plant-to-plant communication. Plant Sci 196:93–100. 10.1016/j.plantsci.2012.08.006 23017903 10.1016/j.plantsci.2012.08.006

[CR77] Zhang MM, Zhang SQ (2022) Mitogen-activated protein kinase cascades in plant signaling. J Integr Plant Biol 64:301–341. 10.1111/jipb.13215 34984829 10.1111/jipb.13215

