# Supplementary material for: Structure-Function Analysis of Volatile (Z)-3-Fatty Alcohols in Tomato
Source: J Chem Ecol. 2025 Jan 24;51(1):6. doi: 10.1007/s10886-025-01557-7 (PMC11761988; doi:10.1007/s10886-025-01557-7)
Supplement: Supplementary file 1 — Supplementary Material 1 [file 10886_2025_1557_MOESM1_ESM.docx]

**STRUCTURE-FUNCTION ANALYSIS OF VOLATILE (*Z*)-3-FATTY ALCOHOLS IN TOMATO**

**Authors:**

^1,2^KIRSTEN FISHER - *ORCID-ID:* *0000-0002-5771-2987*

^1^HARSHITA NEGI - *ORCID-ID:* 0000-0002-9418-5098

^1^OWEN COLE

^1^FALLON TOMLIN

^3^QIAN WANG - *ORCID-ID: 0000-0002-2149-384X*

*^1^JOHANNES W. STRATMANN - *ORCID-ID: 0000-0002-5673-9272*

*Affiliations*

^1^*Department of Biological Sciences, University of South Carolina, Columbia, SC, USA*

^2^*present address: Department of Bacteriology, University of Wisconsin, Madison, Madison, WI, USA*

^3^*Department of Chemistry and Biochemistry, University of South Carolina, Columbia, SC, USA*

**Corresponding author, e-mail: johstrat@biol.sc.edu*

*Tel.: (US)-803-777-5730*


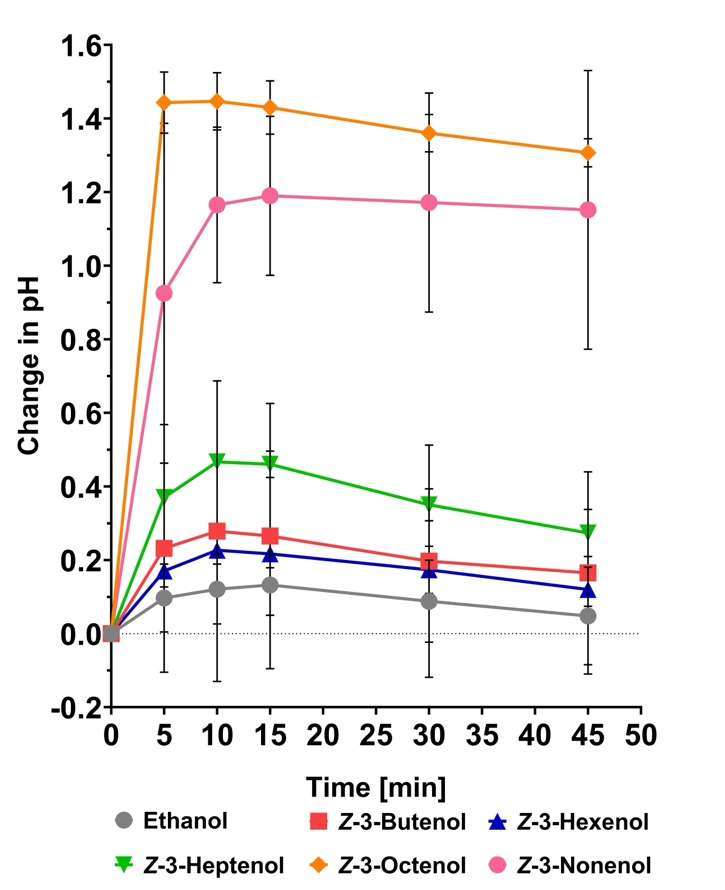


**Supplementary Figure 1** Kinetics of medium pH changes in SP cells elicited by (*Z*)-3-FAlcs at a concentration of 5 mM. (Left) SP cells were treated with 5 mM of (*Z*)-3-FAlcs solved in 10 μL ethanol, and 10 μL ethanol alone. The change in medium pH is expressed as compared to the pH at time = 0. Graphs represent the average and standard deviations of three independent experiments (n=3). (Right) To determine significant differences between treatments, a two‐way ANOVA followed by a Holm‐Šídák multiple comparisons test was performed. Numbers highlighted in green show P-values < 0.05 for significantly different effects of specific (*Z*)-3-FAlcs compared to ethanol and other FAlcs.

**Supplementary Table 1** Statistics for **Fig. 1 (A-F)**. Two‐way ANOVA comparing (*Z*)-3-FAlcs to ethanol control treatments, followed by a Holm‐Šídák multiple comparisons test. Green highlights show P-values < 0.05 for significantly different effects of FAlcs compared to ethanol.

**Supplementary Table 2** Statistics for **Fig. 2 (A,B,C)**. Two‐way ANOVA comparing 1-hexanol, (*E*)-2-hexenol, and (*Z*)-3-hexenol treatments to ethanol control treatments, followed by a Holm‐Šídák multiple comparisons test. Green highlights show P-values < 0.05 for significantly different effects of FAlcs compared to ethanol. See Fig. 2 for Creative Commons statement for the previously published data for (*Z*)-3-hexenol.


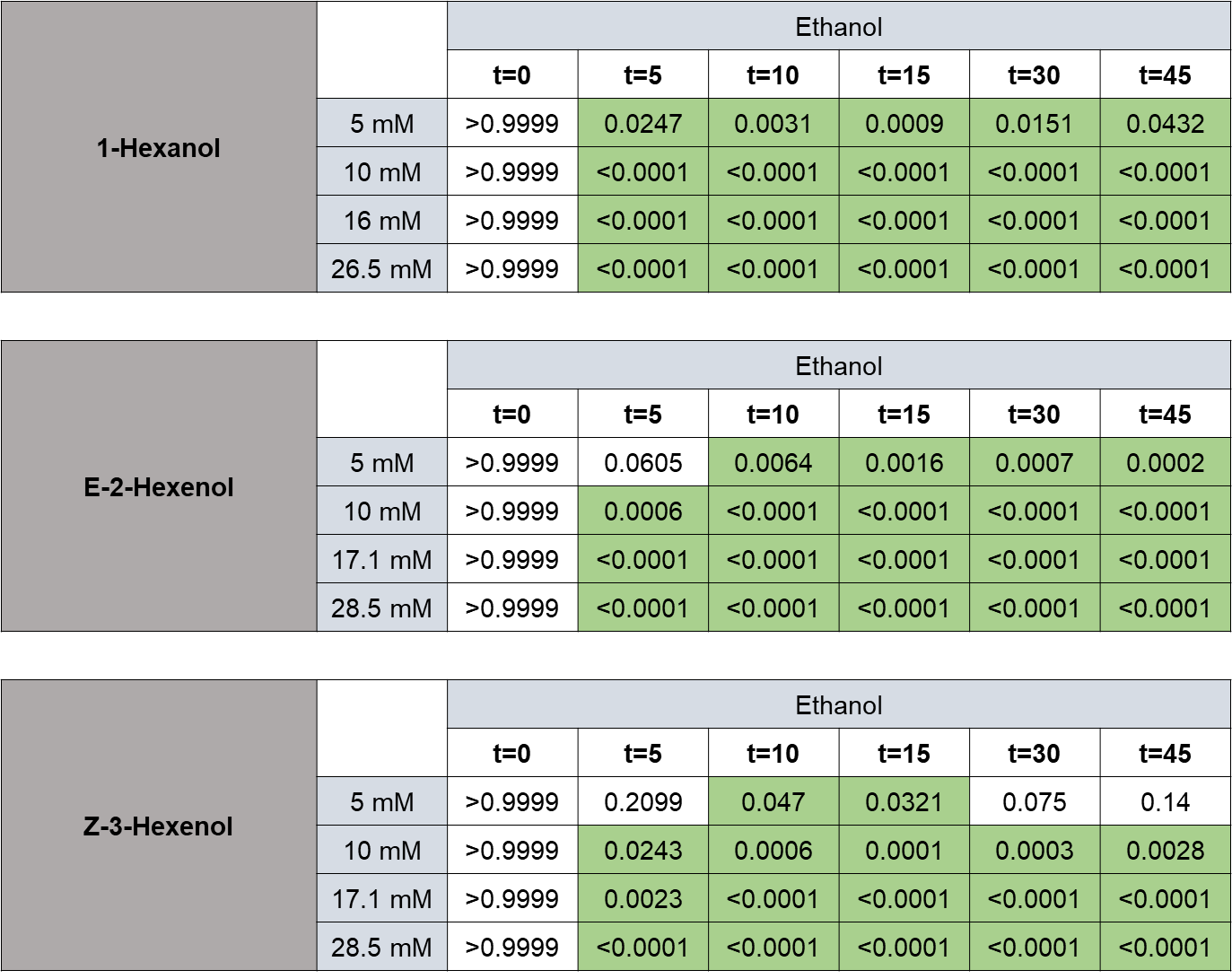


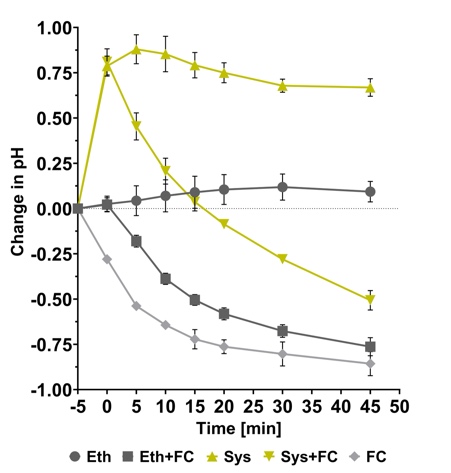


**Supplementary Figure 2** Fusicoccin (FC) induces medium acidification after an initial alkalinization response induced by systemin. SP cells were treated with either 10 µL ethanol or 3 nM systemin at t = -5 minutes or left untreated. Five minutes later (t = 0), cells were either treated with 2.5 μM FC or not, and the medium pH was recorded. The error bars represent the average ± SD of three independent experiments.

**Supplementary Table 3**. Statistics for **Fig. 3 (A-F)**. Two‐way ANOVA followed by a Holm‐Šídák multiple comparisons test comparing the effects of FAlc treatments to double treatments with FAlcs and fusicoccin. Green highlights show P-values < 0.05 for significantly different effects.

**Supplementary Figure 3** Comparison of MAPK phosphorylation in response to (*Z*)-3-FAlcs in three independent experiments. SP cells were treated for 5 – 45 min with 5 mM (*Z*)-3-butenol, -hexenol, -heptenol, and -octenol (Z3-4-OL to Z3-9-OL) that were solved in 10 µL ethanol, and with 10 µL ethanol (Eth) alone. Lane 2 was left empty. Phosphorylation of MAPKs was visualized by immunoblotting using an antibody against the phosphorylated MAPK activation motif pTEpY. Coomassie-stained membranes are shown to demonstrate equal loading and transfer. All three independent experiments are shown. Numbers on bottom of coomassie-stained membranes show pixel intensity on X-ray film obtained with ImageJ. To compare different time points and experiments quantitatively, signals were normalized for each blot, setting the response to octenol as 100% and expressing the response to ethanol and the other (*Z*)-3-FAlcs as a percentage thereof. Showing averages for all three experiments is not informative due to normal variation between experiments and limits for quantification using pixel density in ImageJ. The quantified data only serve to compare (*Z*)-3-FAlc responses per time point and were used to determine how a response to a (*Z*)-3-FAlc compares to the response to octenol. This is expressed as the number of experiments showing a specific response pattern in Fig. 4.

**Supplementary Figure 4** MAPK phosphorylation induced by 5 to 1 mM (*Z*)-3-octenol and (*Z*)-3-nonenol in three independent experiments. SP cells were treated for 5 and 10 min with either 5 mM, 2.5 mM, or 1 mM of (*Z*)-3-octenol or (*Z*)-3-nonenol, or with 10 µL ethanol (solvent). Phosphorylation of MAPKs at 5 and 10 min after treatments was detected as described for Fig. 4 A. Three independent experiments, including coomassie-stained membranes, are shown. Numbers at bottom show number of experiments for each treatment showing a positive MPK1/2 or MPK3 phosphorylation signal.
